# Structural, functional, and stability change predictions in human telomerase upon specific point mutations

**DOI:** 10.1038/s41598-019-45206-y

**Published:** 2019-06-18

**Authors:** U. Kalathiya, M. Padariya, M. Baginski

**Affiliations:** 1 Department of Pharmaceutical Technology and Biochemistry, Faculty of Chemistry, Gdansk University of Technology, Gdansk, Poland; 2 Present Address: International Centre for Cancer Vaccine Science; University of Gdansk, Gdansk, Poland

**Keywords:** Computational models, Computational biophysics

## Abstract

Overexpression of telomerase is one of the hallmarks of human cancer. Telomerase is important for maintaining the integrity of the ends of chromosomes, which are called telomeres. A growing number of human disease syndromes are associated with organ failure caused by mutations in telomerase (hTERT or hTR). Mutations in telomerase lead to telomere shortening by decreasing the stability of the telomerase complex, reducing its accumulation, or directly affecting its enzymatic activity. In this work, potential human telomerase mutations were identified by a systematic computational approach. Moreover, molecular docking methods were used to predict the effects of these mutations on the affinity of certain ligands (C_9i, C_9k, 16A, and NSC749234). The C_9k inhibitor had the best binding affinity for wild-type (WT) telomerase. Moreover, C_9i and C_9k had improved interactions with human telomerase in most of the mutant models. The R631 and Y717 residues of WT telomerase formed interactions with all studied ligands and these interactions were also commonly found in most of the mutant models. Residues forming stable interactions with ligands in molecular dynamics (MD) were traced, and the MD simulations showed that the C_9k ligand formed different conformations with WT telomerase than the C_9i ligand.

## Introduction

Telomerase, a ribonucleoprotein (RNP), acts as a reverse transcriptase that synthesises telomeric DNA repeats at the ends of chromosomes^1^. Telomerase is active in the early stages of life, maintaining the telomere length, and it becomes inactive in most somatic cells during adulthood^2,3^. The ability of a telomere to provide genomic stability decreases over time, owing to both the natural loss of telomeric structure with each cell division (the end replication problem) and the loss of telomerase activity. In addition, this process leads to ageing^4,5^. However, in cancer cells, telomerase becomes reactivated, and it continuously maintains the short length of telomeres in rapidly dividing cells, leading to their immortality^6,7^. Most cancers have adapted mechanisms to protect lengths of telomeres, and this protection is achieved by telomerase activation in approximately 90% of human cancers. Therefore, the role of telomerase in cancer and ageing makes it an important target for cancer therapies and age-associated disorders^8–11^. Additionally, telomere-mediated disorders, such as dyskeratosis congenita, aplastic anaemia, and idiopathic pulmonary fibrosis, have telomerase mutations^12–14^.

Telomerases from evolutionarily distant organisms share a conserved structural organisation^15^, which contains a template-encoding RNA (telomerase RNA or hTR, TER) and a primary protein component (telomerase reverse transcriptase, TERT) with various functional domains (telomerase essential N-terminal domain, TEN; telomerase RNA-binding domain, TRBD; reverse transcriptase domain, RT; and C-terminal extension, CTE)^16–19^. Telomerase-specific domains at the N- and C-terminals of TERTs are not present in any of the viral reverse transcriptases^20^. The N-terminal domain is required for enzymatic function in the assembly of protein with its integral RNA component and in homodimerisation of the protein^20–23^. The C-terminal domain is required for telomerase-specific activity other than catalytic function and in the telomeric nucleotide addition process^24,25^. The fact that mutations of some key residues in telomerase, which are known to affect the conventional reverse transcriptase catalytic activity, also negatively influence telomerase activity strongly argues that the TERT domain is the catalytic domain of the enzyme complex^25–28^. Inherited mutations in both human TERC (the template region of an integral RNA component) and human TERT part of protein lead to rare bone marrow failure syndromes, autosomal dominant dyskeratosis congenita, and acquired aplastic anaemia^28–31^. Many experiments have shown the effects of specific amino acid substitutions or deletions on the enzymatic activity of telomerase and have provided useful knowledge about key residues. In addition, an analysis of such single-residue substitutions and their functional consequences at the molecular level using a complete structural model of telomerase^19^ will be a useful complement to such structural data.

**Figure 1:**
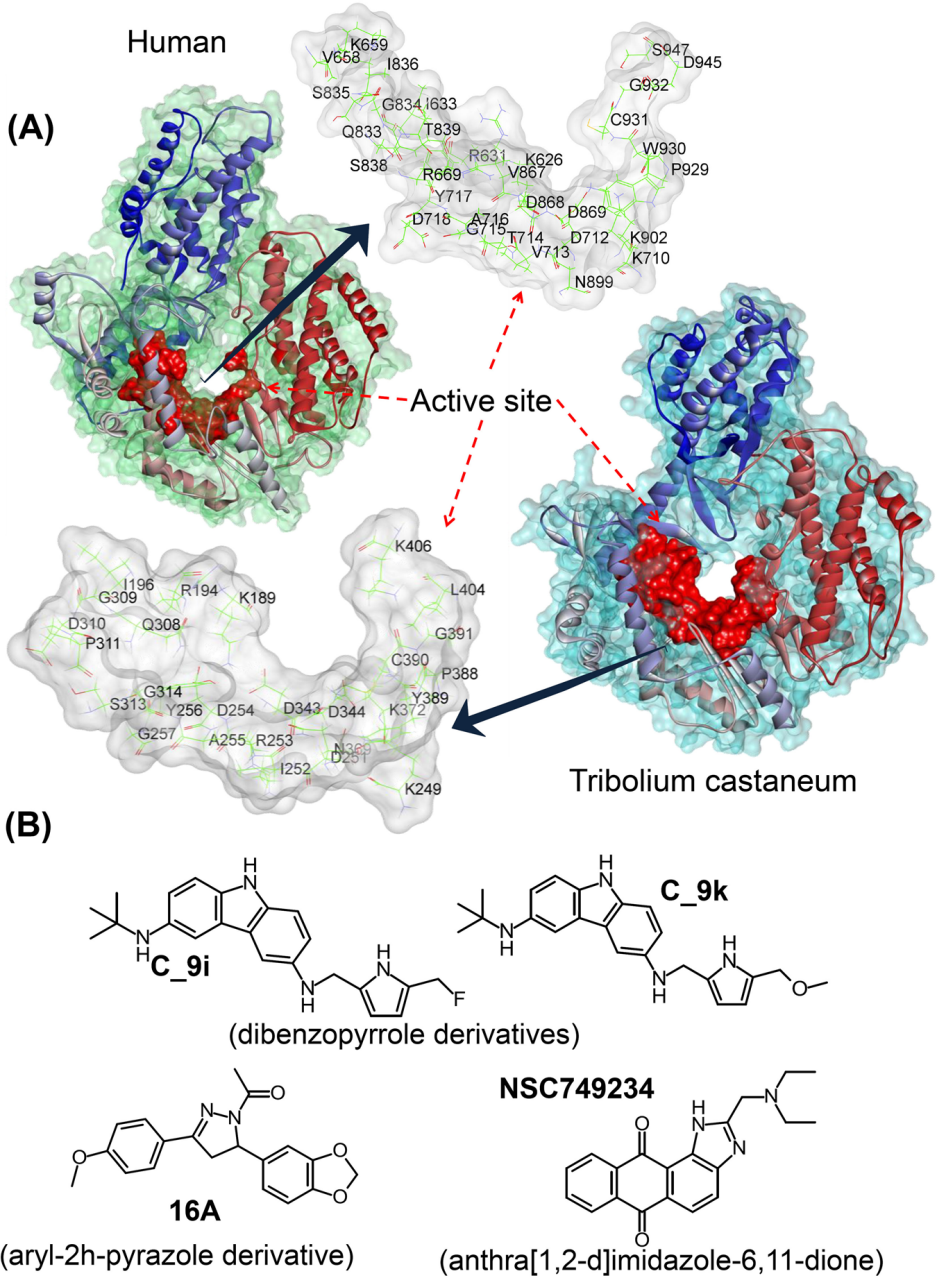
(**A**) The structure of the catalytic subunit (TERT) and active-site residues in *Tribolium castaneum* (PDB: 3DU6)^11^ and the human^19^ telomerase model. (**B**) The structure of ligand molecules, namely, C_9i^33^, C_9k^33^, 16A^34^, and NSC749234^36^, which were selected to study with human telomerase.

The key active-site residues in the TERT catalytic subunit of *Tribolium castaneum* telomerase have been published by Gillis *et al*. in^11^ (PBD: 3DU6; Fig. 1A). Steczkiewicz *et al*.^19^ provided the first structural model of human telomerase, using computational methods derived from the X-ray structure of the full-length *T.castaneum* telomerase (PDB: 3DU6; apo form)^11^ and the enzyme in complex with a RNA∶DNA hairpin (PDB: 3KYL)^32^. In the present study, we used the TERT catalytic subunit of the human telomerase model^19^ and defined/predicted the active-site residues based on *T. castaneum* telomerase structure^11,33–35^ (Fig. 1A). Moreover, residues of telomerase involved in biological functions were also covered in active site^19^. By superimposing the human telomerase structure over *T. castaneum* structure, we identified the active-site residues at the same structural location in both structures. Active site residues of *T. castaneum*/human telomerase were as follows: K189/K626, R194/R631, I196/I633, K249/K710, D251/D712, I252/V713, R253/T714, D254/G715, A255/A716, Y256/Y717, G257/D718, Q308/Q833, G309/G834, D310/S835, P311/I836, S313/S838, G314/T839, D343/D868, D344/D869, N369/N899, K372/K902, P388/P929, Y389/W930, C390/C931, G391/G932, L404/D945, and K406/S947^11,19^. Moreover, residues V658, K659, R669, V867, R972, and K973 of human model were also selected in the current study because previous studies have shown the importance of these residues in different biological functions of human telomerase (Fig. 1A)^19^.

To the best of our knowledge, there have been limited theoretical studies on telomerase that analyse the effect of the mutations at the molecular level. Here, we studied different potential mutations of telomerase enzyme and their effects when binding to various ligands (known as potential inhibitors) by means of molecular docking and molecular dynamics (MD) simulations. The present study provided valuable insights into the nature of potential structural changes as a result of mutations, especially at the functionally important regions or residues of the active site.

Four recently designed or identified telomerase inhibitors, namely, C_9i^33^, C_9k^33^, 16A^34^, and NSC749234^36^, were selected to study with the mutated human telomerase model (Fig. 1B). The C_9i and C_9k compounds are derivatives of dibenzopyrrole, and *in silico* analysis of this new chemical scaffold has shown potential telomerase-binding properties^33^. Compound 16A^34^ is from a series of novel aryl-2h-pyrazole derivatives containing an oxygen-bearing heterocyclic group. Compound 16A has potent inhibition activity for telomerase and good activity against human melanoma cell B16-F10^34^. The NSC749234 compound^36^ is a derivative of anthra[1,2-d]imidazole-6,11-dione and has been evaluated for telomerase inhibition, hTERT expression and suppression of cancer cell growth *in vitro*^36^.

To study potential mutations of residues in active-site of human telomerase, two different approaches were followed. For the first approach, the Blast tool implemented in the UniProtKB/Swiss-Prot^37^ database was used to search natural mutants in proteins (having similar sequences) other than telomerase. This approach takes advantage of the fact that evolution has been performing its own massively parallel mutagenesis and selection experiments over time. Thus, these natural sequence variations found in other proteins can be more likely to occur also in telomerase active site. In the second approach, the mutant (MT) series was compared against wild-type (WT) using the Molecular Operating Environment (MOE; Chemical Computing Group Inc.), and for a particular residue all possible point mutations were searched to determine mutational consequences on telomerase^38^. Mutations that have shown similar behaviour with all four ligands as well as those obtained the highest and lowest energies compared to WT telomerase were studied by molecular docking to reveal potential differences in ligand-binding properties. Furthermore, all-atomic-level studies were performed by MD simulations to better understand the protein dynamics and the effects of the mutations on protein structure.

## Materials and Methods

### Structural model of human telomerase

Steczkiewicz *et al*.^19^ employed a theoretical modelling approach to generate the entire 3D structure of the human TERT, TEN, and TRBD bound to a DNA substrate and its RNA template. They used an advanced meta-profile comparison method, Meta-BASIC^39^, to map the human telomerase protein sequence onto sequences of the determined structures from Tribolium and Tetrahymena. The mappings obtained were confirmed by using a variety of fold recognition methods. Steczkiewicz *et al*.^19^ built three-dimensional models separately for TEN and the other components of the human telomerase complex including a hybrid RNA∶ DNA double helix formed between the RNA template and the single-stranded telomeric DNA substrate and assembled them by using protein-protein docking, guided by relevant experimental data (Fig. S1A). In this study, we used the TERT catalytic subunit of the human telomerase model provided by Steczkiewicz *et al*.^19^.

### Selection of natural variants

Short sequences (containing active-site residues) of human telomerase were submitted to the Blast tool implemented in UniProtKB/Swiss-Prot^37^ to identify variations in similar sequences (natural mutants). Residues that were predicted as natural mutants in protein other than telomerase and mutations occurring with unusual frequency in different organisms were selected for the present study. From the screening results, four residues, namely, R631, Y717, D868, and D869, were considered because they have shown frequent mutations or variants in other organisms (Fig. S2). In total, seven natural mutant models, namely, R631Q, R631P, Y717G, D868N, D868K, D869R, and D869S, were generated. Model structures of human telomerase with novel natural mutations were built with the help of the ‘Built Mutant’ protocol incorporated in the modelling environment of the Discovery Studio Client v18.1 programme [Dassault Systemes, BIOVIA Corp., San Diego, CA, USA]. The ‘Built Mutant’ protocol uses the Modeler programme (version: 9v8)^40^ to mutate residues to specified types and optimises the conformation of both the mutated residues and neighbouring residues. Side chains of the residues in the models were further refined to correct their conformation and orientation using the ‘Chi Rotor’ modelling programme^41^.

The generated natural mutant models and ligand structures were energy minimised with default parameters of the ‘smart minimiser’ algorithm applying CHARMm forcefield^42^ in the Discovery Studio Client v18.1 programme. Following energy minimisation, flexible molecular docking was performed using CDOCKER, a CHARMm-based docking tool^43^ from Discovery Studio Client v18.1, to analyse ligand affinity towards different mutated human telomerase models. In CDOCKER, 100 random ligand conformations were generated in the active site of the telomerase structure. The temperature was set to 700 K for 2000 steps and cooled to 300 K for 5000 steps. The grid extension was set to 8 Å, and 10 ligand binding poses were ranked according to their CDOCKER energies.

### MOE point mutations

The ‘Sequence Design’ protocol of the protein design module incorporated in MOE, which calculates frequency and probability of amino acids at residue mutation sites (mutation expression), was used in the present study, and a ligand affinity score was obtained when ligand binds to WT and mutated telomerase (point mutation). The goal of this method is to modulate physical protein properties such as stability or affinity. Sequence Design is meant to handle situations where the “combinatorial explosion” due to the number of residues and mutations reaches a point where producing all mutants is not feasible due to sterical hindrances. In addition, for this method the wild type mutation is always allowed to compete since in some cases, if no better mutant is possible, the best mutant will be simply the wild type^44^. This approach was followed for four different telomerase-ligand complexes, in which all 33 functional residues were mutated by 20 amino acids [A, R, N, D, C, Q, E, G, H, I, L, K, M, F, P, S, T, W, Y, and V]. In each point mutation, the rotamer explorer RMSD limit was set to 0.25 Å, energy window to 10 kcal/mol and residues farther than 4.5 Å were kept fixed. LowMode MD, the search method in MOE, was used with default parameters for a thorough conformational search because it is a recent and particularly relevant addition to the MOE toolbox for a wide set of structures from small molecules to peptides, macrocycles, and protein loops. The CHARMM27 force field was used, and the maximum number of search iterations was 10000. Mutation analysis in MOE showed that mutation of 16 residues from 33 functional residues affected ligand affinity. Furthermore, considering these results, mutations of these 16 residues were further studied by the CDOCKER docking programme in Discovery Studio Client v18.1.

### Docking of WT human telomerase

Protein-ligand binding affinity was computed using the CDOCKER docking programme for WT human telomerase when in complex with four different ligands, using the same parameters used for the docking of natural mutants. Furthermore, rigid and induced fit (flexible) dockings were performed using the MOE software package^44^. In MOE, all four complexes were subjected to energy minimisation using the CHARMM27 forcefield, and docking was performed with the ‘Rigid Receptor’ and ‘Induced Fit’ docking protocols. One thousand ligand conformations were generated, and the docking was performed using the ‘Triangle Matcher’ placement method, which is the most efficient method for well-defined binding sites. All 1000 conformations per ligand were scored using the ‘London dG’ scoring function, submitted to a refinement step based on molecular mechanics and rescored with the ‘GBVI/WSA dG’ scoring function^45,46^. GBVI/WSA dG, a forcefield-based scoring function, determines the binding free energy (kcal/mol) of the ligand from a given pose^45,46^.

### Molecular dynamics

MD simulations were performed for eleven different systems as follows: (i) WT human telomerase in apo-form; (ii) and (iii) WT human telomerase with ligand C_9i and C_9k (CDOCKER binding mode); (iv) and (v) WT human telomerase with ligand C_9i and C_9k (MOE rigid binding mode); (vi) and (vii) WT human telomerase with ligand C_9i and C_9k (MOE induced fit binding mode); (viii) and (ix) mutated Y717H human telomerase with C_9i and C_9k (CDOCKER binding mode); and (x) and (xi) mutated Y717R human telomerase with C_9i and C_9k (CDOCKER binding mode). All simulations were performed with the GROMACS 4.6.5 simulation suite^47^ using the Gromos96 43a1 forcefield parameter set^48^. The PRODRG server was used for generating topologies of ligands that can be used in GROMACS^49^. The SPC water condition was selected, and the box was defined as a dodecahedron. Periodic boundary conditions were applied in all directions, and Na^+^Cl^−^ counter ions (yielding a 150 mM NaCl solution) were added to neutralise the system. The system was energy minimised (50,000 steps of steepest descents) to relax any steric conflicts. Energy minimisation was then followed by NPT equilibration for 1000 ps (1 ns). The particle mesh Ewald method^50^ was employed to account for the long-range electrostatic interactions, and the LINCS algorithm^51^ was used to restrain bond lengths. To maintain constant temperature and pressure (300 K and 1 bar), the V-rescale thermostat^52^ and Parrinello-Rahman barostat^53^ were used, respectively. The production run was performed for 100 ns with leapfrog integrator^54^, and the coordinates were saved every 10 ps.

Ligand binding free energy was calculated using Molecular Mechanics/Poisson-Boltzmann Surface Area (MM/PBSA) method^55^. Binding energy of each snapshot was calculated for each complex using g_mmpbsa tool of Gromacs^56^. The entropy contribution was not included in the binding energy. Principal component analysis (PCA) was carried out to obtain a mass-weighted covariance matrix of the protein atom displacement that is indicative of dominant and collective modes of the protein from the overall dynamics of the MD trajectory. The covariance matrix was diagonalized to extract a set of eigenvectors and eigenvalues that reflect concerted motion of the molecule^57^. To yield the eigenvalues and eigenvectors by calculating and diagonalizing the covariance matrix, Gromacs in-built tool g_covar was used. To analyze and plot the eigenvectors, the g_anaeig tool was used^58^.

## Results and Discussion

### WT human telomerase-ligand binding

**Figure 2:**
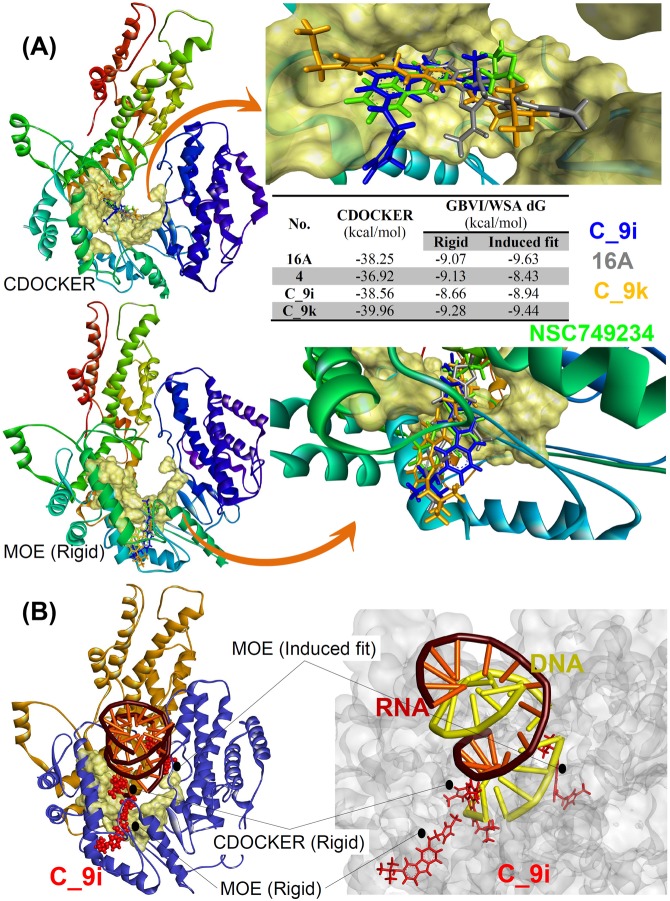
(**A**) Interaction energies and binding of the C_9i, C_9k, 16A, and NSC749234 inhibitors in the active site of WT human telomerase according to CDOCKER and MOE rigid (GBVI/WSA dG) docking. The active site is shown as a surface model, and the inhibitor is shown as a stick model. (**B**) Binding modes of compound C_9i according to CDOCKER, MOE induced fit and rigid docking. In all, C_9i binds close to DNA binding region.

Before analysing the effect of mutations on ligand binding at the structural level, we examined the binding affinity of four different inhibitors (C_9i, C_9k, 16A, and NSC749234) for the native (WT) protein. For the WT *human* telomerase model, both flexible and rigid docking were performed to enhance the sampling space of protein-ligand interactions. The C_9k inhibitor had the best docking score in flexible (CDOCKER) and rigid (MOE) docking, and it had a good binding score in flexible docking of MOE relative to other compounds (Fig. 2).

The docking analysis of WT telomerase showed that Arg631 and Tyr717 residues formed potential interactions with all four ligands and that the Asp868 residue also formed interactions with all ligands, except compound 16A, in docking studies of CDOCKER (Fig. S3). In rigid docking of MOE, Arg865 was dominant and formed interactions with all ligands, except ligand C_9k. Moreover, Arg669 formed interactions with two different ligands, namely, NSC749234 and C_9i (Fig. S4). In MOE flexible or induced fit docking, the Val997, Ile1004, and Asn571 residues interacted most efficiently with the 16A and C_9i ligands, respectively (Fig. S5). Superposition of all four compounds from rigid docking showed that all studied ligands occupied the same binding cleft in the human telomerase model structure (Fig. 2).

The similar binding mode of the ligand in different docking programs is not always the best binding affinity conformation. Here also, top ranked binding complexes from different docking programs have shown different binding mode of the compound C_9i with human telomerase. However, in top ranked pose from all docking programs, ligand C_9i occupies the regions near to the DNA binding site (Fig. 2B). All compounds were also able to form same binding mode in different docking program though with not highest affinity. One of the example is shown for compound C_9i from MOE Induced fit and Rigid docking (Fig. S1B).

### Mutated human telomerase interactions

By applying the MOE point mutations approach, the ligand affinity score was computed for the different possible mutations. From the 33 active-site residues, mutations of 16 residues (Lys626, Arg631, lys710, Asp712, Ala716, Tyr717, Asp718, Gln833, Thr839, V867, Asp868, Asp869, Lys902, Pro929, Asp945, and Ser947) resulted in changes in the ligand affinity (Fig. S6, Tables S1 and S2). On contrary, other studied mutations of the 14 other residues (Ile633, V658, K659, R669, Val713, Thr714, Ser835, Ile836, Ser838, Asn899, Trp930, Cys931, R972, and K973) had no effect on the ligand affinity score, showing that these residues were far from the ligand-binding region. Moreover, for three residues, namely Gly715, Gly834, and Gly932, mutations were not possible in the MOE point mutations approach.

Mutations of the K626, K710, T839, D868, and P929 residues in the presence of ligand 16A consistently obtained higher affinity scores than for the WT, whereas any variants of R631 had lowered affinity compared to the WT. Mutations of the A716, Y717, D718, and V867 residues did not show much effect on ligand affinity. Most of the mutations of D712, Q833, K902, D945, and S947 obtained almost similar ligand affinity scores with a few exceptions having higher affinity than for WT (D712Q, Q833R, K902R, D945R, S947W, S947Y, S947R, and S947K). Among the point mutations of D869, the D869A and D869G mutations had considerably decreased affinity for ligand 16A, while the other mutations obtained almost similar scores (Tables S1 and S2). For the NSC749234 ligand, mutations of the R631, T839, and D868 residues always obtained higher affinity scores than for the WT, whereas any mutation of Y717, Q833, and D869 had lower affinity than for the WT. The A716, D718, and V867 variants did not show considerable effects in ligand affinity. Most of the mutants of D712, P929, D945, S947, K626, K710, and K902 had similar ligand affinity scores with some exceptions having higher/lower affinity (D712C, P929R, P929K, D945R, S947W, S947F, S947Y, S947R, S947K, K626Q, K626N, K710F, K710A, K710C, K710N, K710S, K710E, K710D, K710G, K902C, K902S, K902N, K902D, and K902H) than WT (Tables S1 and S2).

Predicted MOE point mutations of K626 and R631 showed reduced ligand affinity compared to WT in the presence of the C_9i ligand, whereas D712, A716, Y717, D718, Q833, T839, V867, D868, and S947 mutants obtained ligand affinity scores almost the same as the wild type mutation. Most of the mutations of K710, K902, P929, and D945 obtained almost the same ligand affinity scores with few exceptions showing considerably higher affinity than WT (K710F, K710R, K902F, K902R, K902Q, K902E, K902H, P929W, P929F, and D945R). Among the D869 mutations, D869A and D869G had considerably lower affinity to the C_9i ligand, while other D869 mutations obtained almost the same scores (Tables S1 and S2). In the case of the C_9k ligand, mutation of T839 was only possible by T839R and obtained a higher affinity score than for WT, whereas mutations of K626, V867, and D868 did not show considerable effects on ligand affinity. Most mutations of R631, K710,D712, A716, Y717, D718, D869, K902, P929, and D945 residues obtained similar ligand affinity scores with a few exceptions having lower/higher affinity (R631S, D712A, A716S, A716N, Y717F, Y717N, Y717D, D869G, S947M, S947R, S947Q, S947E,K710F, K710Y, K710M, K710R, K710Q, D718L, K902R, K902Q, P929N, D945W, S947W, S947F, S947Y, and S947K) than WT (Tables S1 and S2).

**Figure 3:**
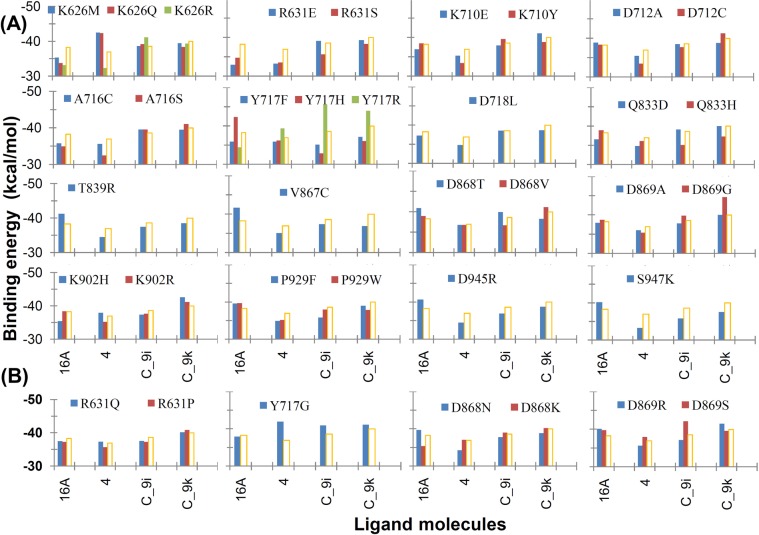
CDOCKER interaction (binding) energy for mutations obtained from (**A**) MOE point mutations approach and (**B**) naturally occurring mutations approach. The orange bar represents the interaction energy for WT telomerase.

**Figure 4:**
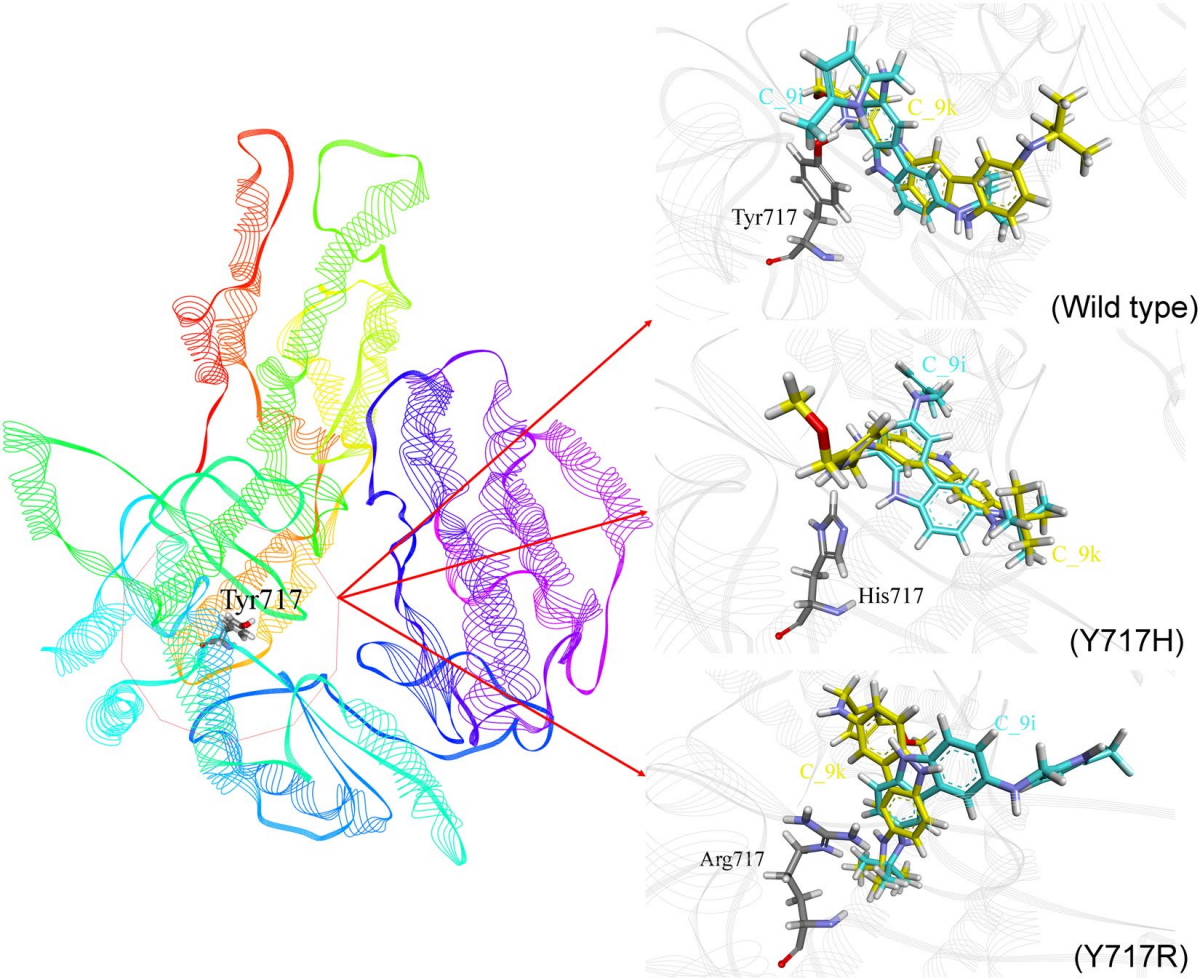
Binding conformation of the C_9i and C_9k ligands with respect to the WT and mutated human telomerase models (Y717H and Y717R) according to CDOCKER docking.

Seven mutant models, namely, R631Q, R631P, Y717G, D868N, D868K, D869R, and D869S, obtained from the naturally occurring mutations approach and 29 mutations (of 16 residues) that obtained considerably higher or lower ligand affinity scores in the MOE mutation approach were validated by the CDOCKER docking programme in Discovery Studio Client v18.1. In CDOCKER docking of four studied ligands, protein-ligand interaction energy and interacting residues were identified for all variants (Figs 3 and 4, Tables S3 and S4). The docking score or interaction energy of the WT telomerase-16A complex was −38.25 kcal/mol, whereas the docking score ranged from −32.91 to −42.27 kcal/mol for the mutant model. Similarly, the docking score was −36.92 kcal/mol for the WT telomerase-NSC749234 complex, and it ranged from −32.37 to −42.50 kcal/mol for the mutant models. For the dibenzopyrrole derivatives (C_9i and C_9k), the docking score to the WT telomerase was −38.56 and −39.96 kcal/mol, respectively. For the mutant models, the docking score of C_9i ranged from −32.83 to −45.53 kcal/mol, and the docking score of C_9k ranged from −36.05 to −44.63 kcal/mol (Tables S3 and S4).

Comparing binding of the dibenzopyrrole derivatives (C_9i and C_9k) with the other two studied inhibitors (16A and NSC749234), the dibenzopyrrole derivatives had improved interactions with telomerase in most of the studied mutant models. Inhibitor 16A showed similar binding in the majority of studied natural mutants. Most of the mutations reduced binding of the NSC749234 inhibitor to telomerase or had similar binding as to the wild type one (Fig. 3). The Arg631 and Tyr717 residues of WT telomerase formed interactions with all four studied ligands and were also found frequently in most of the mutant models. The Asp868 residue in WT model formed interactions with all ligands, except 16A. Compound 16A formed interactions with Cys931 and Gly932, and compound C_9i formed an interaction with Gln833 in the WT telomerase (Table S3).

Work by Drosopoulos and Prasad, showed that substitutions at Val867 lead to significant changes in overall enzyme activity and telomere repeat extension rate, but have little effect on polymerase processivity. And also indicated that Val867 plays a key role in nucleotide incorporation and suggested that this residue may provide important interactions with incoming substrate nucleotides^59^. In our analysis, mutations of V867 does not affect the ligand affinity of compound C_9i and C_9k.

Previous studies suggest that three conserved uncharged residues Y717, Q833, and V867 form a hydrophobic pocket adjacent to the catalytic aspartates and take part in nucleotide binding^19,32^. Residue mutation at V867 has been shown to alter human telomerase substrate specificity, and at Q833, mutations cause hypersensitivity to substrate analogs^19,59,60^. In our docking analysis, WT telomerase residue Tyr717 formed potential interactions with all four ligands. Moreover, mutations of all three residues Tyr717, Gln833, and Val867 resulted some changes in the ligand affinity.

Docking analysis of the WT and mutant models indicated the effect of mutation on the ligand binding efficiency. The overall analysis suggested that two novel mutations, namely, Y717H and Y717R, showed considerable effects on binding affinity. The Y717H mutant reduced the interaction energy, and the Y717R mutant improved the binding towards dibenzopyrrole derivatives (C_9i and C_9k) compared to other variants. Moreover, both C_9i and C_9k derivatives obtained conformations or binding modes similar to WT telomerase, whereas they changed their conformation in the Y717H and Y717R mutant models, which resulted in lower and higher binding affinity, respectively (Fig. 4).Considering the impact of these mutations on the binding affinity, mutated Y717H and Y717R human telomerase models with C_9i and C_9k were selected to investigate the alteration in structural-dynamics properties by the MD approach.

### MD simulations

MD simulations were performed for WT telomerase in the apo-form and in complex with either C_9i or C_9k ligand. The top ranked binding affinity pose of compound C_9i and C_9k have obtained different binding mode in CDOCKER, MOE rigid and induced fit docking. Considering this, simulations were performed for each of the binding mode taken as the starting structure for the MD (Fig. S7). In addition, MD was performed on two mutated human telomerase models, namely, Y717H and Y717R, with the respective C_9i and C_9k ligands. For mutant models, the best docked conformation of both ligands into the active site of human telomerase obtained from CDOCKER was taken as the starting structure for the MD run.

**Figure 5:**
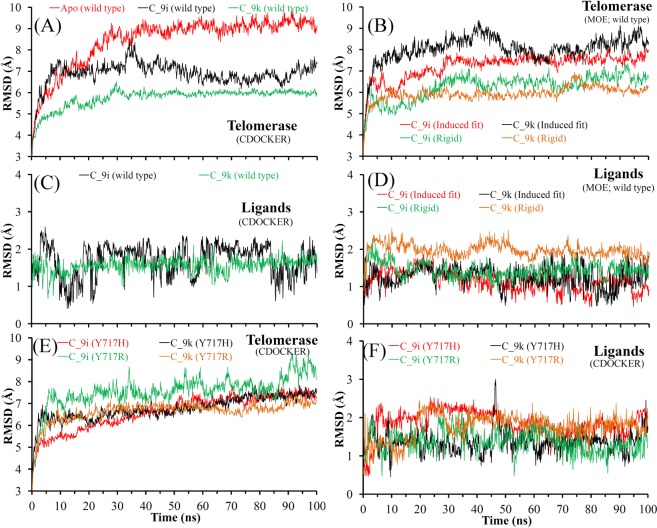
RMSDs of (**A**) WT telomerase in apo-form and in complex with C_9i/C_9k from MD of CDOCKER binding mode, (**B**) WT telomerase in complex with C_9i/C_9k from MD of MOE induced fit or rigid binding mode, (**C**,**D**) ligand C_9i, C_9k from MD of CDOCKER and MOE induced fit or rigid binding mode, respectively, (**E**,**F**) mutated human telomerase (Y717H and Y717R) and ligand molecules from the complex (MD of CDOCKER binding mode). RMSDs were computed for all atoms, excluding hydrogens.

The two most common measures of structural fluctuations, root-mean-square deviation (RMSD) and root-mean-square fluctuation (RMSF), were analysed for all simulated systems. As shown in Fig. 5A, WT telomerase had high RMSD values in the apo-form compared to when in complex with the C_9i or C_9k ligand. The RMSD analysis of MD simulations starting from the CDOCKER and MOE rigid docking poses suggested more stable telomerase structure (with lower RMSDs) when in complex with the C_9k ligand than with the C_9i ligand. The reverse was observed for MOE induced fit docking poses, telomerase structure has obtained lower RMSDs when in complex with the C_9i ligand than with the C_9k ligand (Fig. 5). The RMSD of telomerase structure in complex with C_9k was higher by approximately 1 Å in both mutant models compared to WT (Fig. 5A,E). In the case of systems with the C_9i ligand, the Y717H mutation had similar RMSDs as for WT telomerase, whereas the Y717R mutation obtained higher RMSDs by 1 to 2 Å (Fig. 5A,E). The RMSDs of both ligands C_9i and C_9k showed similar behaviour (~2 Å) in all simulated systems (Fig. 5).

**Figure 6:**
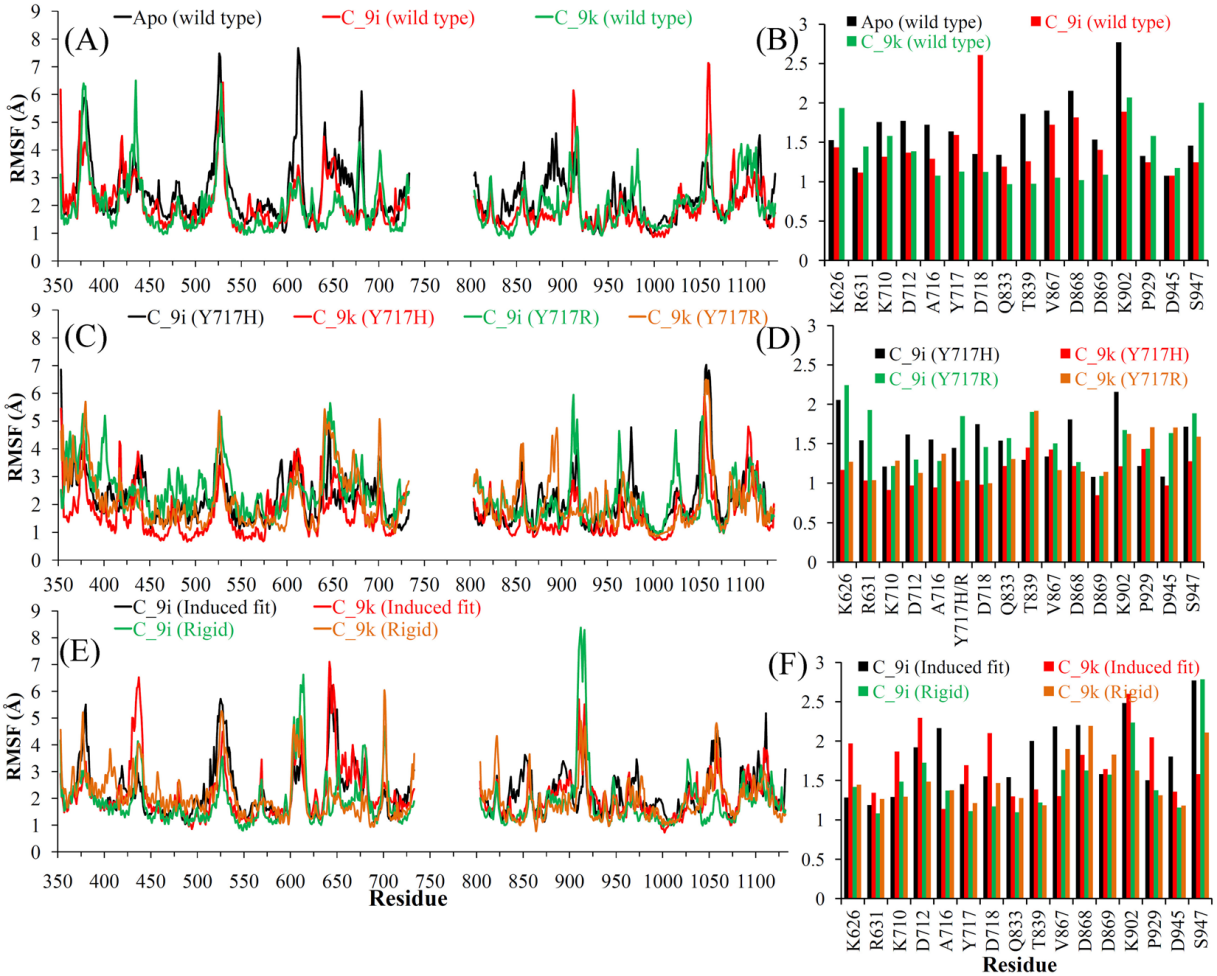
Residue-based RMSF of (**A**,**B**) WT telomerase in apo-form and in complex with C_9i/C_9k from MD of CDOCKER binding mode, (**C**,**D**) mutated human telomerase (Y717H and Y717R) in complex with C_9i/C_9k from MD of CDOCKER binding mode, (**E**,**F**) WT telomerase in complex with C_9i/C_9k from MD of MOE induced fit or rigid binding mode. RMSFs were computed for Cα atoms.

These findings suggested that structural fluctuations of WT human telomerase were found less pronounced in the presence of ligand compared to the apo-form, and similar behaviour was observed in 16 specific residues with only a few exceptions (for which mutations affected the ligand affinity; Figs 3 and 6). Overall the RMSF analysis of mutated models with the C_9i and C_9k ligands showed similar fluctuations in all four systems. However, the RMSFs of the 16 specific residues indicated that there were fewer structural fluctuations for the mutated models in complex with the C_9k ligand compared to the C_9i ligand (Fig. 6).

Hydrogen bond (H-bond) interactions between human telomerase and ligands (C_9i and C_9k) were analysed. The residues forming relatively stable contacts with the ligands were identified by taking into account the occupancy time (>1%). The residues forming H-bond interactions in MD simulations starting from the CDOCKER poses are given in Table S5. Asp868, a single residue, formed conserved interactions with the C_9i ligand in all the simulated models. The H-bond interactions of the Thr567 and Gly834 ligand with the C_9i ligand were common to the WT and Y717H mutated models. Moreover, the interaction between the Gln569 residue and the C_9i ligand in WT telomerase was the same as the Y717R mutated model. For the C_9k ligand, the Gln833 and Thr839 interacting residues were common to both Y717H and Y717R mutated models. In addition, the Asp868 residue formed an interaction with the C_9k ligand in the WT and Y717R models (Table S5). Additionally, the residues forming H-bond interactions in MD simulations starting from the MOE rigid and induced fit docking poses are given in Table S6.

**Figure 7:**
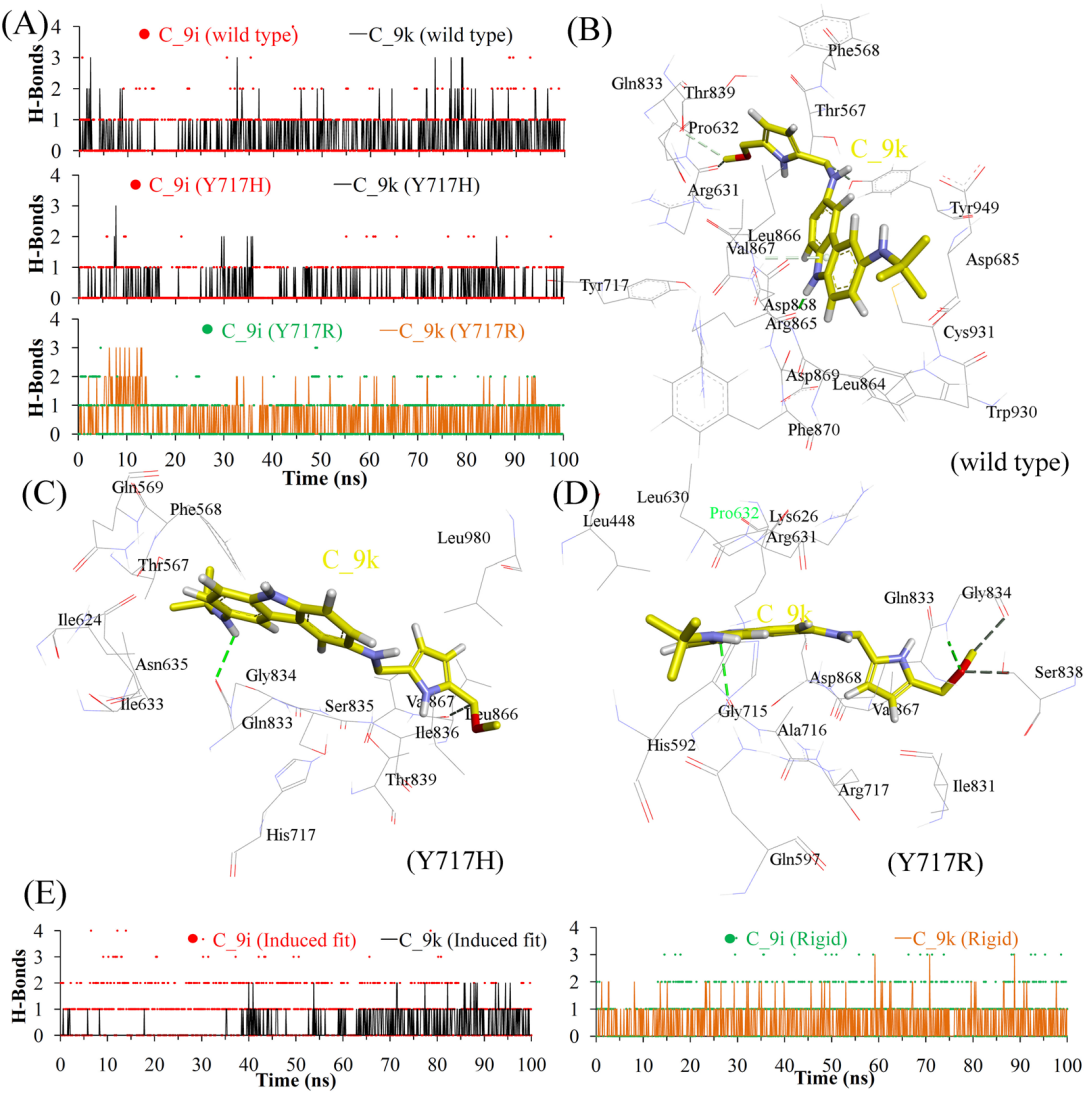
H-bond interactions of human telomerase with inhibitors (C_9i and C_9k). (**A**) Number of intermolecular H-bonds between the protein and ligand from MD of CDOCKER binding mode. (**B**–**D**) Binding pattern of the C_9k ligand in the WT as well as Y717H and Y717R mutant models from MD of CDOCKER binding mode. (**E**) Number of intermolecular H-bonds between the protein and ligand from MD of MOE induced fit or rigid binding mode.

The analysis of H-bond statistics suggested that both the C_9i and C_9k ligands showed more interactions with the WT human telomerase model compared to the studied mutant models (Y717H and Y717R). The complex of ligand with WT telomerase model was stabilised by two to three H-bond interactions, whereas in the mutant models, the ligand mostly formed one to two H-bonds (Fig. 7). The residues of the human telomerase model interacting with the inhibitors (C_9i or C_9k) and the binding mode of inhibitors at the active site of WT and mutant models were traced and are presented in Fig. 7. Additionally, 2D interaction diagrams showing different interactions were further analysed for all simulated models and are shown in Fig. S8.

**Figure 8:**
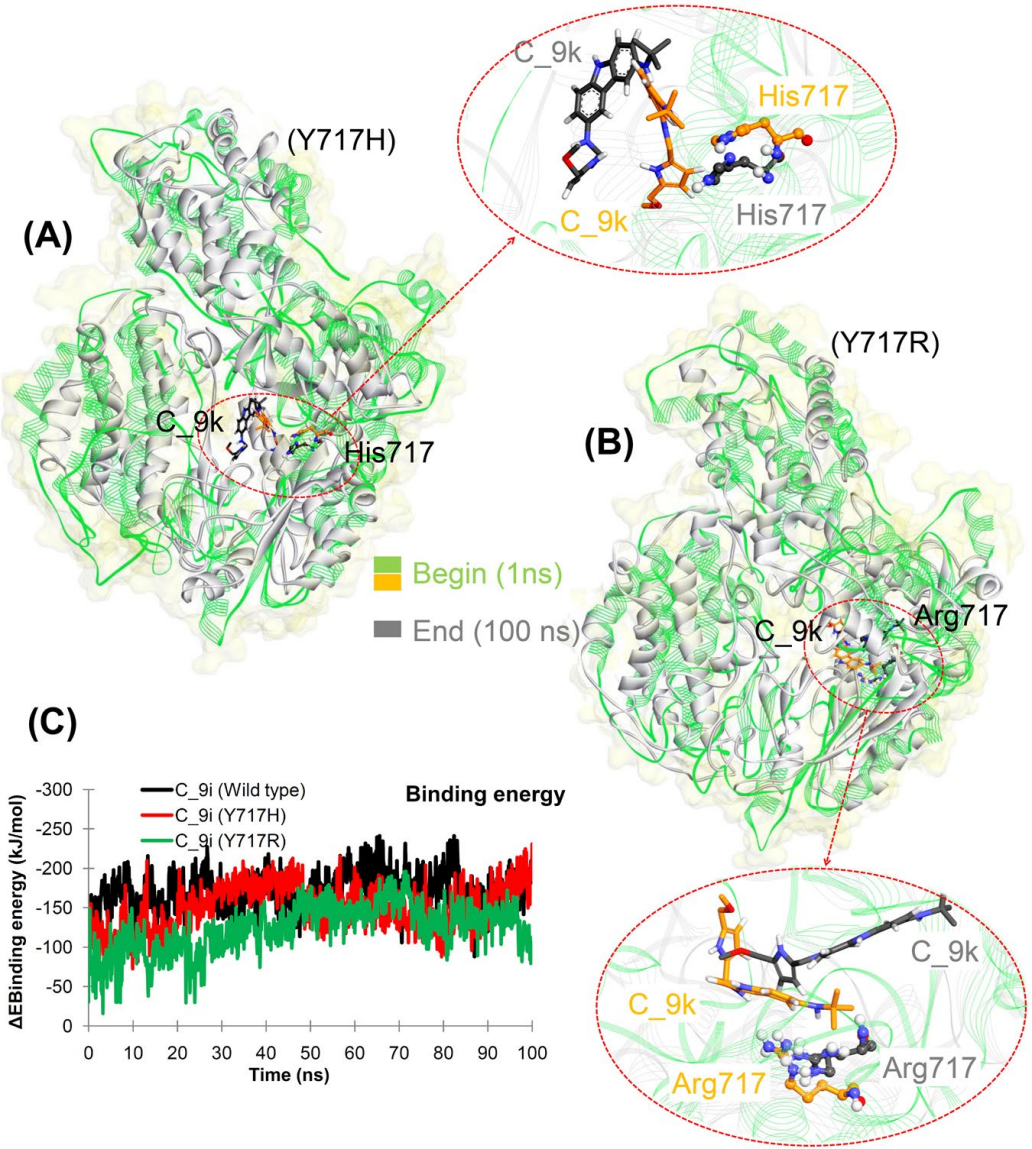
Overlay of the binding mode of the C_9k inhibitor at the beginning and at the end of the MD simulations of (CDOCKER binding mode), (**A**) Y717H and (**B**) Y717R mutant models. (**C**) Calculated MM-PBSA binding energy of compound C_9i with telomerase (from MD of CDOCKER binding mode).

To trace the dynamics of ligand binding to the human telomerase model, simulated structures of WT and mutant models were analysed from the beginning and end of the MD simulations (Figs 8, S9 and S10). Both the C_9i and C_9k ligands showed similar binding modes with WT telomerase in docking, whereas the C_9k ligand had different conformations in the MD simulation of CDOCKER binding mode (Figs 4 and S9). For the mutant models, conformation analysis of the C_9i and C_9k ligands from the MD simulations correlated well with the docking findings. As shown in Fig. 8, the C_9k ligands had less conformational change in the Y717R model than in the Y717H model, which correlated with the observation from the docking that C_9k had a higher binding affinity in the Y717R model compared to the Y717H model. In contrast, the C_9i ligand showed different conformations in the Y717R model compared to those in the Y717H model in the MD simulation, and the findings were similar to the docking results (Figs 4 and S10).

To better estimate binding energy than just taking into account scores from docking programs, we performed MM-PBSA calculations taking into account MD trajectories. The analysis was performed three protein systems, namely WT and mutated telomerase (at Y717H or Y717R) in complex with ligand C_9i. Comparative analysis can be performed using the obtained MM-PBSA analysis since the entropic contributions were not included in the binding energy. As seen in Fig. 8C, ligand C_9i obtained the best binding energy when in complex with WT telomerase and results also indicated that C_9i may behave similarly in Y717H mutated complex. Lower affinity of this ligand was recorded towards the second mutant Y717R. This observation correlates well with the energy contribution from the van der Waals, electrostatic, polar, and non-polar interactions (Fig. S11A). Furthermore, a quantitative assessment of the binding energy at individual residue had been studied as well (Fig. S11B). The residues with high contribution are placed in the binding region.

In order to analyse potential movement of residues in the binding region, the principal component analysis of MD trajectory was performed. This analysis was performed for WT human telomerase and mutant model Y717H with ligand C_9i. Presentation of significant motions projected along first eigenvector shows that introduced mutation Y717H drive the ligand movement. Due to that ligand motions, the movement of protein fragments in telomerase active site was observed. In WT human telomerase bound with C_9i, no such protein motions were observed relative to the mutant model (Figs S12 and S13).

## Conclusions

The human telomerase model structure was screened for the prediction of natural variants, and a MOE point mutations protocol was applied to obtain a large-scale structural analysis of the mutations. WT and variants of human telomerase were designed to study the binding mode of the ligands (C_9i, C_9k, 16A, and NSC749234) by molecular docking and MD simulations. The C_9k inhibitor preferentially binds to WT human telomerase compared to the other compounds. Site-directed mutation (point mutations) obtained by MOE studies pointed to mutations of 16 residues that may influence ligand affinity towards human telomerase (K626, R631, K710, D712, A716, Y717, D718, Q833, T839, V867, D868, D869, K902, P929, D945, and S947). Compared to the 16A and NSC749234 ligands, the dibenzopyrrole derivatives (C_9i and C_9k) had improved their interactions with human telomerase in most of the studied mutant models. The R631 and Y717 residues of WT telomerase formed interactions with all four studied ligands and were also found frequently in most of the mutant models. In the MD studies, the D868 residue formed stable H-bond interactions with the C_9i ligand in all simulated models, and the T567 and G834 residues interacting with C_9i were the same in the WT and Y717H mutated models. Moreover, an interaction between Q569 and C_9i was the same in the Y717R and WT telomerase models. The Q833 and T839 residues interacting with the C_9k ligand were the same in both mutated models (Y717H and Y717R). D868 formed interactions with the C_9k ligand in the WT and Y717R models. In molecular docking, both the C_9i and C_9k ligands showed similar binding modes with WT telomerase, whereas the C_9k ligand showed different conformations in the MD simulations. The detailed large-scale structural analysis of mutations/variants in human telomerase significantly enhances the predictive power of existing approaches that can be utilised in the development of new/improved potent inhibitors against telomerase.

## Supplementary information

Supporting Materials
