# Supplementary material for: Structural, functional, and stability change predictions in human telomerase upon specific point mutations
Source: Sci Rep. 2019 Jun 18;9:8707. doi: 10.1038/s41598-019-45206-y (PMC6581908; doi:10.1038/s41598-019-45206-y)
Supplement: Supplementary file 1 — Supporting Materials [file 41598_2019_45206_MOESM1_ESM.pdf]

Structural, functional, and stability change predictions in human telomerase upon specific point mutations

U. Kalathiya[1,2]*, M. Padariya[1], & M. Baginski[1]

[1]*Department of Pharmaceutical Technology and Biochemistry, Faculty of Chemistry, Gdansk University of Technology, Gdansk, Poland.*

[2]*International Centre for Cancer Vaccine Science; University of Gdansk, Gdansk, Poland (current affiliation).*

**Correspondence and requests for materials should be addressed to U.K. (email: umesh.kalathiya@pg.edu.pl, umesh.kalathiya@ug.edu.pl)*

SUPPORTING MATERIALS

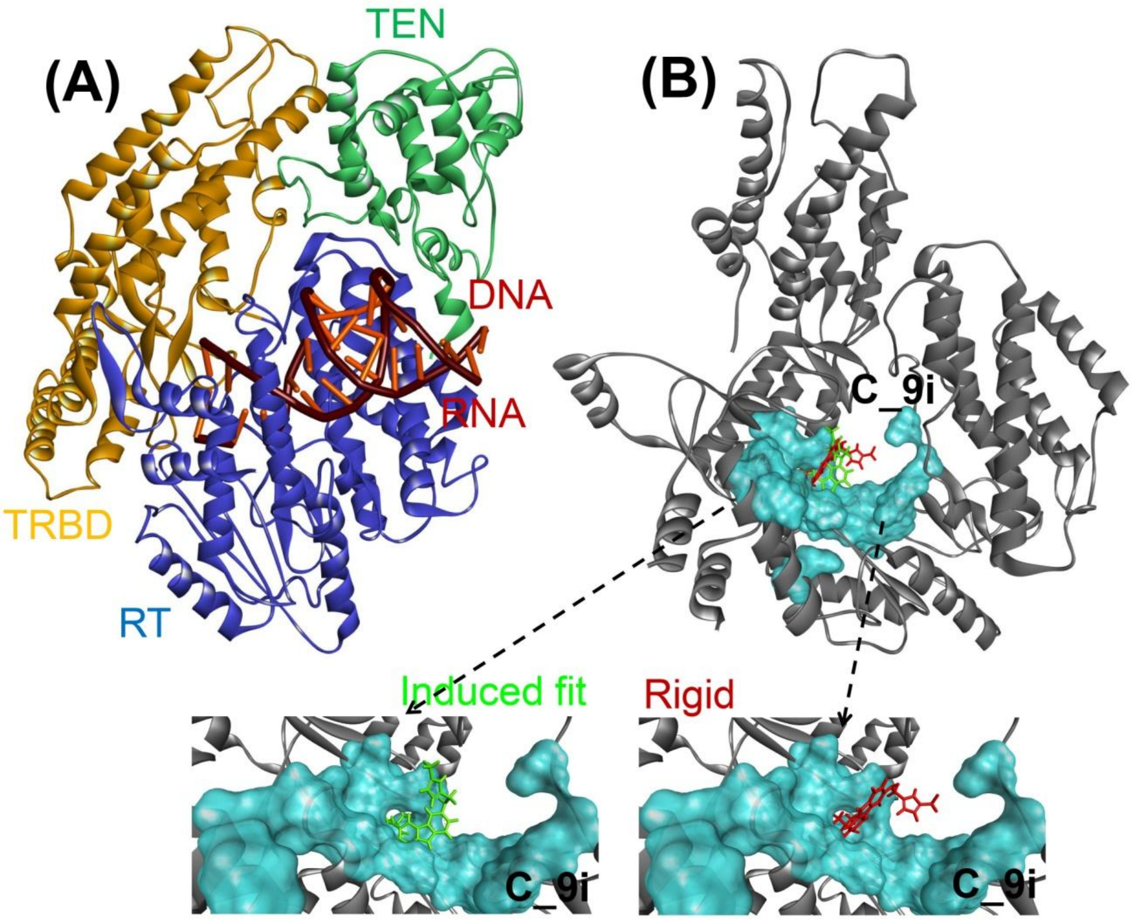

Figure S1. (A) The human telomerase model built by Steczkiewicz et al. [2011] includes the TERT protein component composed of the catalytic reverse transcriptase domain (RT), the RNA binding domain (TRBD), and the N-terminal "anchor" domain (TEN), bound to the template-encoding RNA and the single-stranded human telomeric DNA substrate. **(B)** Binding of compound C_9i in MOE rigid (-8.59 kcal/mol, rank = 3) and induced fit (-8.73 kcal/mol, rank = 4) docking showing similar mode of interaction to that in CDOCKER docking (Figure 2A).

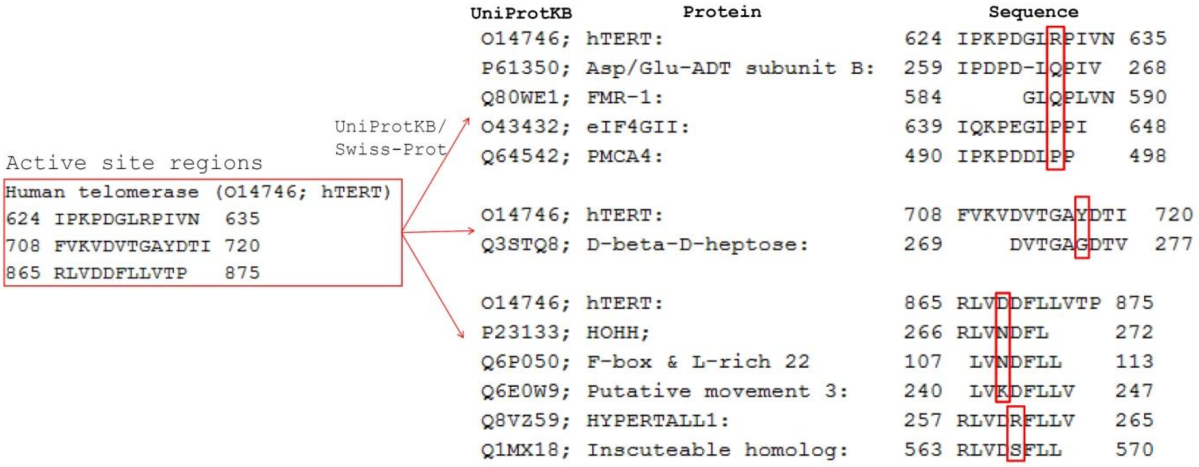

Figure S2. Short sequences (containing active-site residues) of human telomerase submitted to the Blast tool implemented in UniProtKB/Swiss-Prot. Result showing natural sequence

variations found with other proteins at residue R631, Y717, D868, and D869 that are more favourable to occur as telomerase mutations.

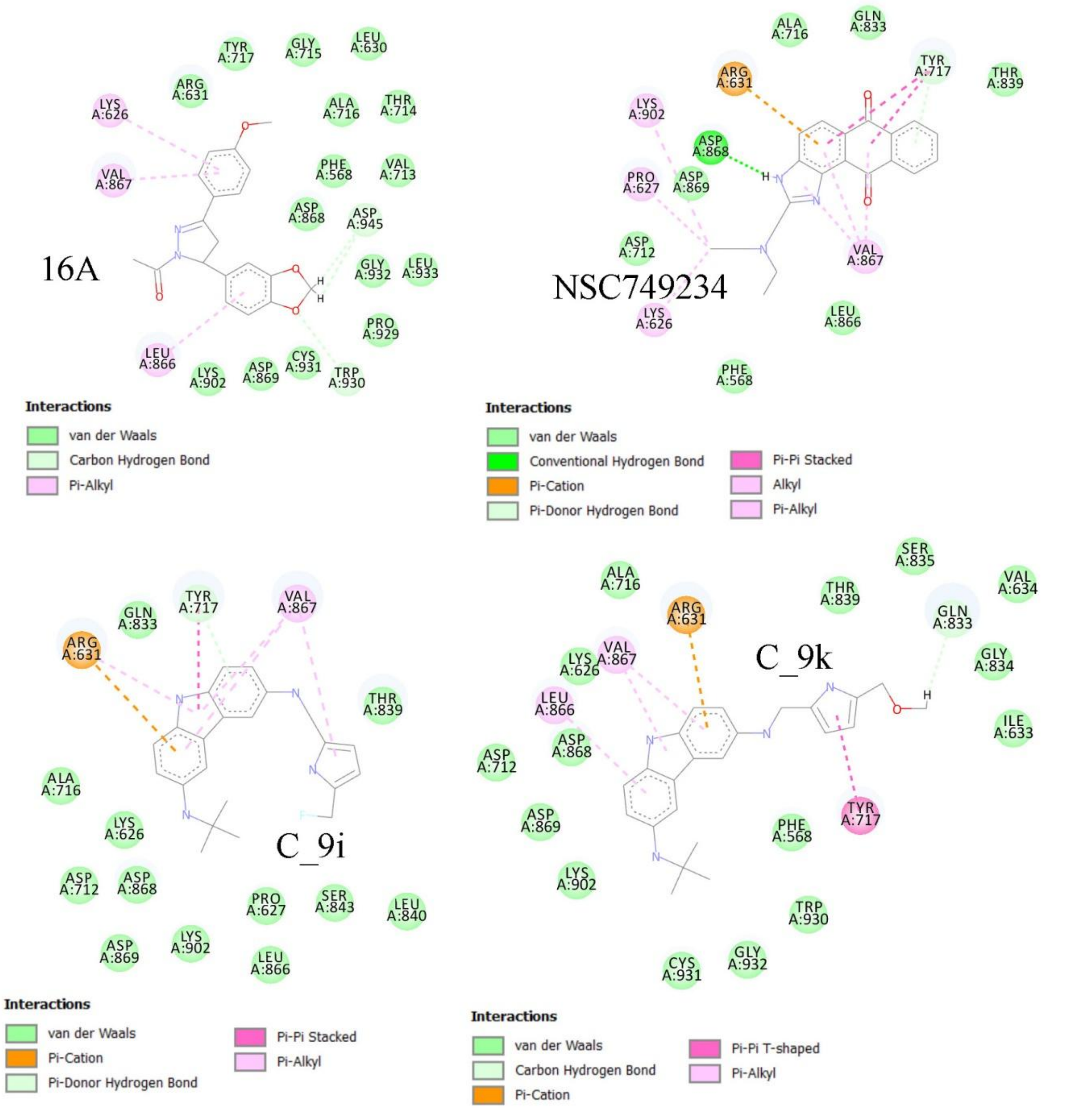

Figure S3. 2D ligand interaction diagram presenting different interactions of the inhibitors C_9i, C_9k, 16A, and 4(NSC749234) with the residues of wild-type (WT) human telomerase obtained from the CDOCKER docking program.

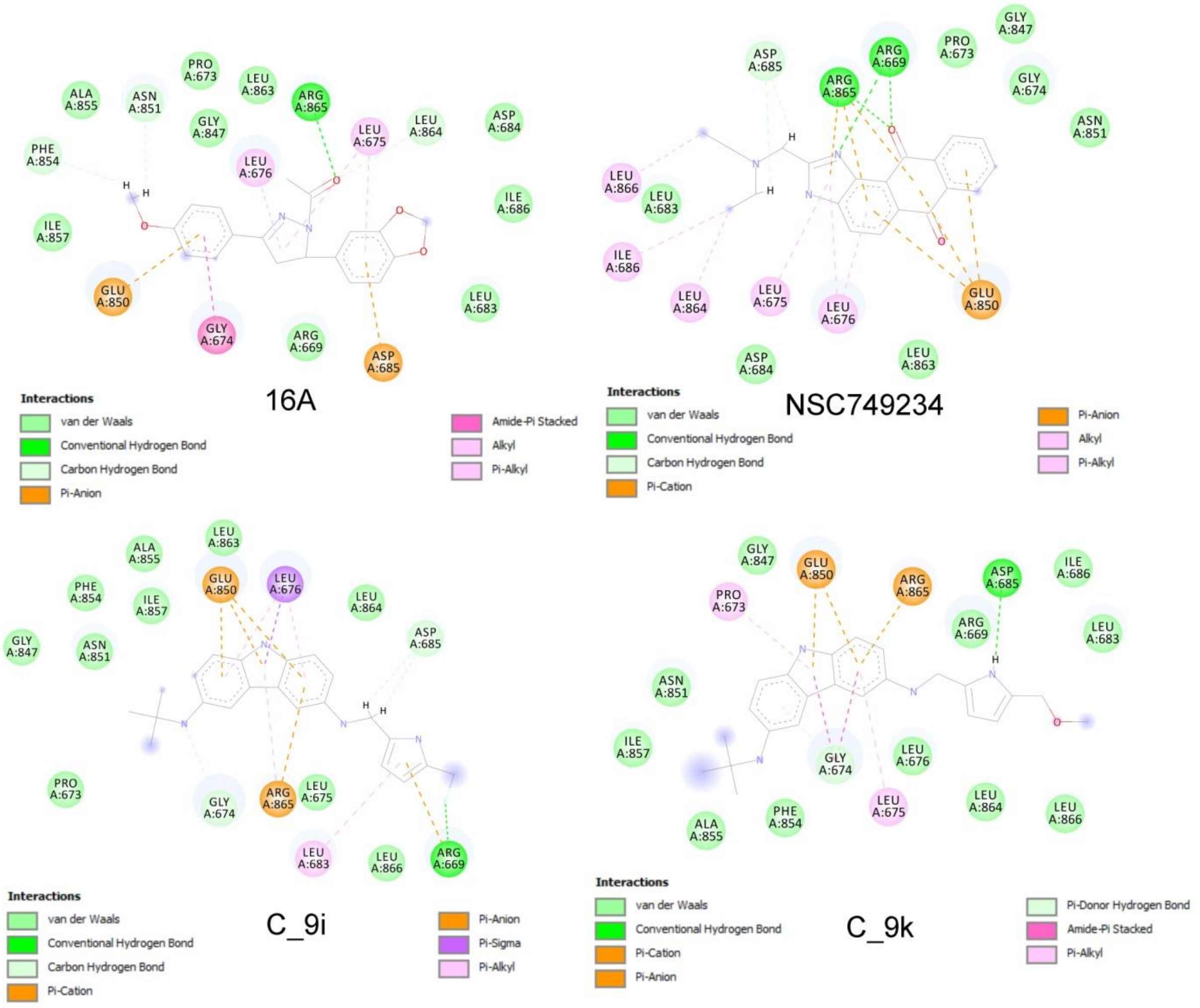

Figure S4. 2D interaction map of the inhibitors C_9i, C_9k, 16A, and 4(NSC749234) with the WT human telomerase obtained from the rigid docking of the MOE software.

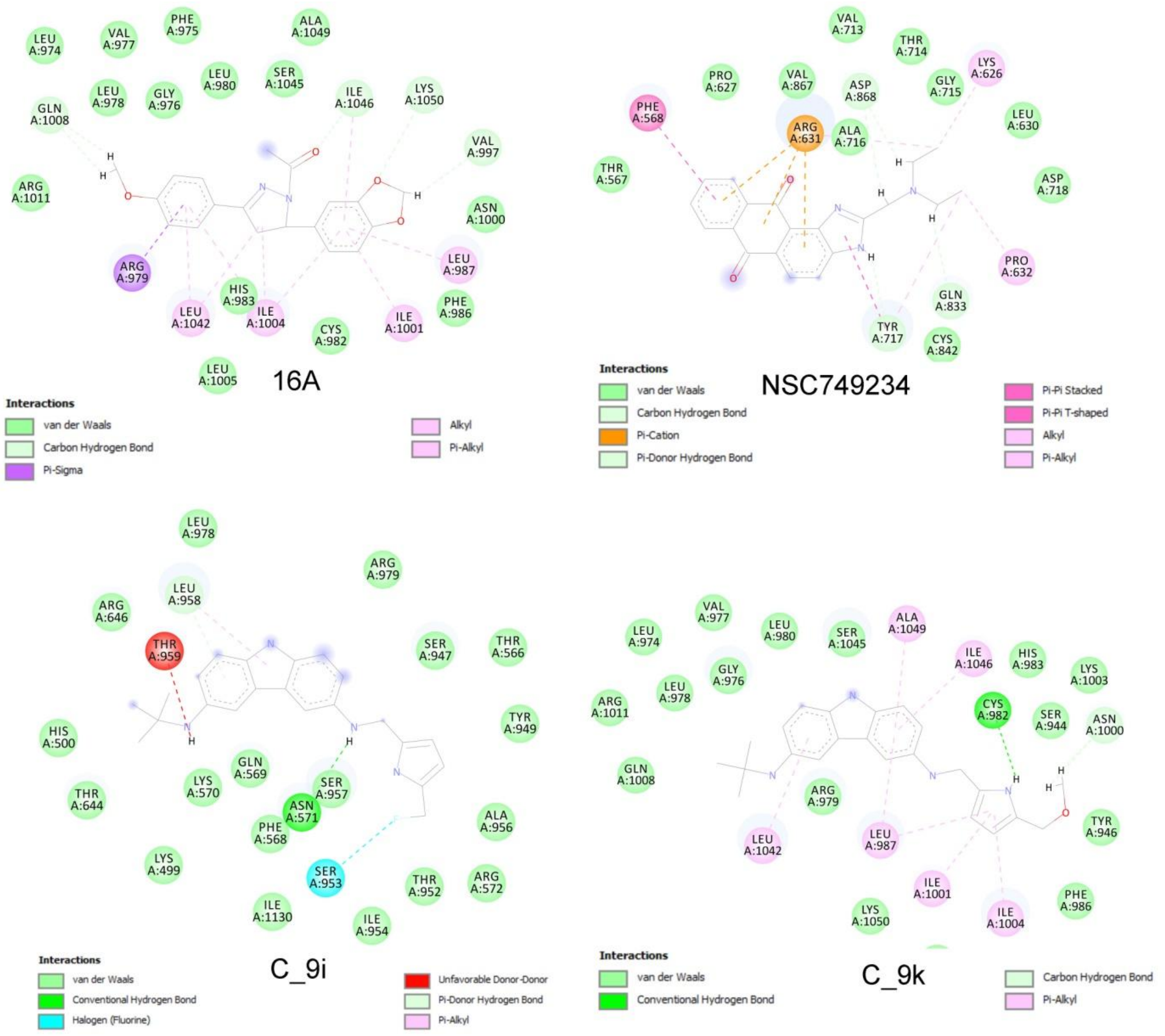

Figure S5. 2D interaction map of the inhibitors C_9i, C_9k, 16A, and 4(NSC749234) with the WT human telomerase obtained from the induced fit docking of the MOE software.

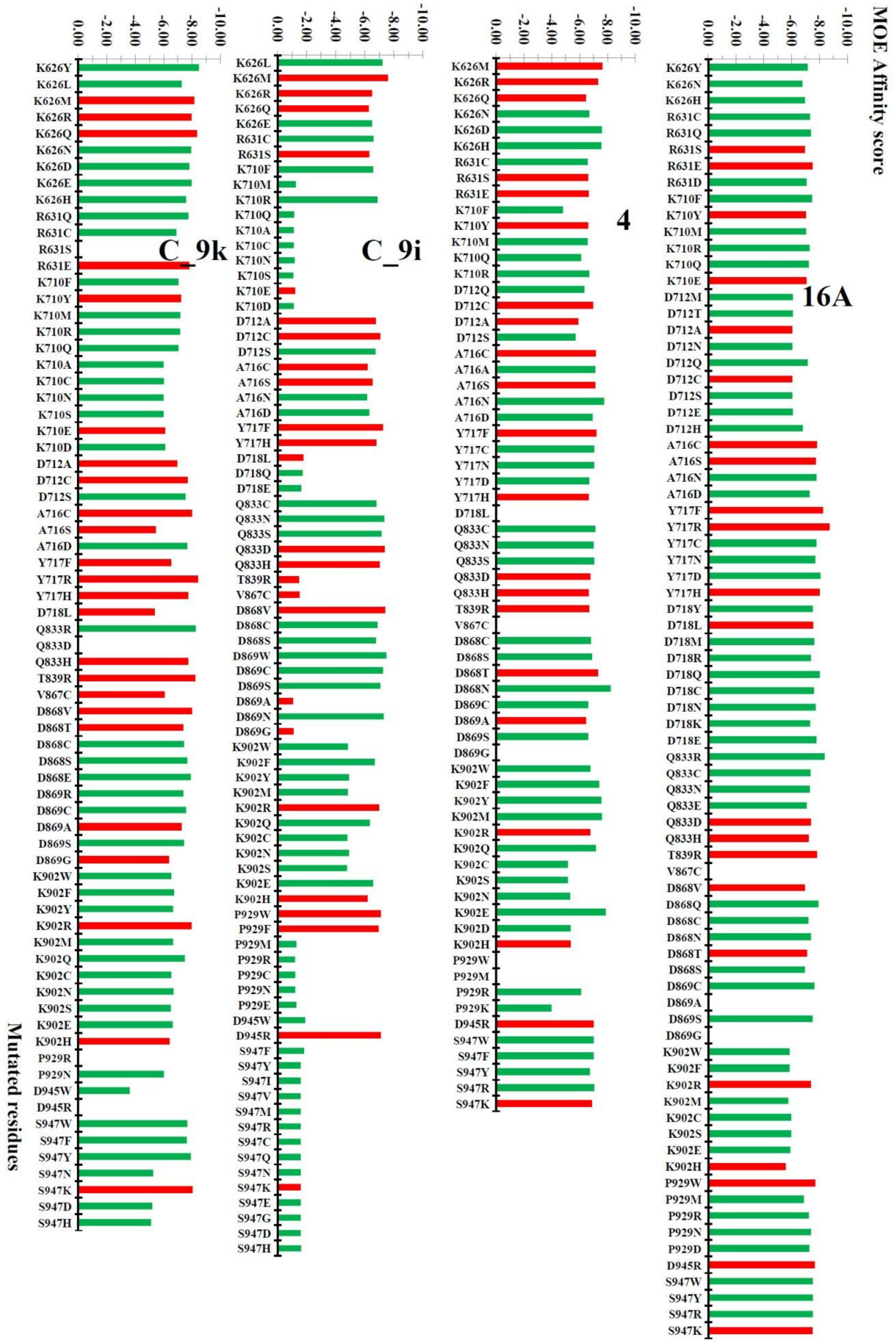

Figure S6. The ligand affinity score (GBVI/WSA dG; kcal/mol) computed for the point mutations obtained from the MOE programme. Mutations of 16 residues (affected ligand affinity) shown in red were selected for further study by the CDOCKER docking program in

Discovery Studio Client v18.1. Mutations having ligand affinity scores that were positive values are listed without any values/bar.

Table S1. The ligand affinity score for the mutations of residues K626, R631, K710, D712, A716, Y717, and D718 obtained from the MOE multiple mutations approach.

mutseq	16A		4		C_9i		C_ 9k	
	mutation	Affinity	mutation	Affinity	mutation	Affinity	mutation	Affinity
**K626**	K626Y	-7.11	K626M	-7.60	K626L	-7.20	K626Y	-8.46
	K626N	-6.77	K626R	-7.32	K626M	-7.56	K626L	-7.25
	K626H	-6.94	K626Q	-6.42	K626R	-6.45	K626M	-8.14
			K626N	-6.65	K626Q	-6.25	K626R	-7.94
			K626D	-7.57	K626E	-6.46	K626Q	-8.32
			K626H	-7.51			K626N	-7.94
							K626D	-7.81
							K626E	-7.98
							K626H	-7.57
**R631**	R631C	-7.31	R631C	-6.58	R631C	-6.60	R631Q	-7.73
	R631Q	-7.34	R631S	-6.60	R631S	-6.29	R631C	-6.90
	R631S	-6.92	R631E	-6.63			R631S	+
	R631E	-7.51					R631E	-7.81
	R631D	-7.05						
**K710**	K710F	-7.45	K710F	-4.77	K710F	-6.57	K710F	-7.03
	K710Y	-7.00	K710Y	-6.60	K710M	-1.24	K710Y	-7.19
	K710M	-7.03	K710M	-6.58	K710R	-6.84	K710M	-7.17
	K710R	-7.27	K710Q	-6.08	K710Q	-1.12	K710R	-7.16
	K710Q	-7.20	K710R	-6.64	K710A	-1.10	K710Q	-7.04
	K710A	+	K710A	+	K710C	-1.10	K710A	-6.00
	K710C	+	K710C	+	K710N	-1.16	K710C	-6.01
	K710N	+	K710N	+	K710S	-1.09	K710N	-6.00
	K710S	+	K710S	+	K710E	-1.19	K710S	-6.00
	K710E	-7.05	K710E	-5.62	K710D	-1.12	K710E	-6.08
	K710D	+	K710D	+			K710D	-6.10
			K710G	+				
**D712**	D712M	-6.06	D712Q	-6.35	D712A	-6.73	D712A	-6.94
	D712T	-6.07	D712C	-6.96	D712C	-7.03	D712C	-7.70
	D712A	-6.03	D712A	-5.88	D712S	-6.72	D712S	-7.54
	D712N	-6.02	D712S	-5.71				
	D712Q	-7.14						
	D712C	-6.01						
	D712S	-6.03						
	D712E	-6.07						
	D712H	-6.78						
**A716**	A716C	-7.80	A716C	-7.15	A716C	-6.17	A716C	-7.99
	A716S	-7.74	A716S	-7.12	A716S	-6.52	A716S	-5.42
	A716N	-7.79	A716N	-7.71	A716N	-6.12	A716N	+
	A716D	-7.28	A716D	-6.91	A716D	-6.30	A716D	-7.64
**Y717**	Y717F	-8.22	Y717F	-7.20	Y717F	-7.23	Y717F	-6.51
	Y717R	-8.72	Y717C	-7.03	Y717H	-6.77	Y717R	-8.40
	Y717C	-7.77	Y717N	-7.04			Y717N	+
	Y717N	-7.67	Y717D	-6.65			Y717D	+
	Y717D	-8.07	Y717H	-6.62			Y717H	-7.71
	Y717H	-7.99						
**D718**	D718Y	-7.51	D718L	+	D718L	-1.74	D718L	-5.38
	D718L	-7.52	D718Q	+	D718Q	-1.72	D718Q	+
	D718M	-7.63	D718N	+	D718E	-1.63	D718N	+
	D718R	-7.37	D718E	+			D718E	+
	D718Q	-7.98						
	D718C	-7.60						
	D718N	-7.71						
	D718K	-7.32						
	D718E	-7.75						

"+" denotes the lower affinity/positive value.

Table S2. The ligand affinity score for the mutations of residues Q833, T839, V867, D868, D869, N899, K902, P929, C931, D945, and S947 obtained from the MOE multiple mutations approach.

mutseq	16A		4		C_ 9i		C_9k	
	mutation	Affinity	mutation	Affinity	mutation	Affinity	mutation	Affinity
**Q833**	Q833R	-8.36	Q833C	-7.09	Q833C	-6.80	Q833R	-8.25
	Q833C	-7.35	Q833N	-7.01	Q833N	-7.35	Q833C	+
	Q833N	-7.29	Q833S	-7.04	Q833S	-7.14	Q833N	+
	Q833E	-7.07	Q833D	-6.74	Q833D	-7.36	Q833S	+
	Q833D	-7.34	Q833H	-6.63	Q833H	-7.01	Q833D	+
	Q833H	-7.22					Q833H	-7.72
**T839**	T839R	-7.80	T839R	-6.68	T839R	-1.45	T839R	-8.22
**V867**	V867C	+	V867C	+	V867C	-1.52	V867C	-6.06
**D868**	D868V	-6.91	D868C	-6.81	D868V	-7.40	D868V	-7.97
	D868Q	-7.92	D868S	-6.85	D868C	-6.86	D868T	-7.36
	D868C	-7.19	D868T	-7.28	D868S	-6.76	D868C	-7.43
	D868N	-7.37	D868N	-8.22			D868S	-7.66
	D868T	-7.10					D868E	-7.90
	D868S	-6.95						
**D869**	D869C	-7.62	D869C	-6.61	D869W	-7.49	D869R	-7.38
	D869A	+	D869A	-6.45	D869C	-7.25	D869C	-7.56
	D869S	-7.47	D869S	-6.59	D869S	-7.07	D869A	-7.25
	D869G	+	D869G	+	D869A	-1.04	D869S	-7.44
					D869N	-7.29	D869G	-6.34
					D869G	-1.06		
**K902**	K902W	-5.83	K902W	-6.74	K902W	-4.80	K902W	-6.53
	K902F	-5.81	K902F	-7.37	K902F	-6.65	K902F	-6.73
	K902R	-7.39	K902Y	-7.56	K902Y	-4.88	K902Y	-6.68
	K902M	-5.76	K902M	-7.58	K902M	-4.82	K902R	-7.95
	K902C	-5.96	K902R	-6.78	K902R	-6.95	K902M	-6.67
	K902S	-5.95	K902Q	-7.16	K902Q	-6.31	K902Q	-7.48
	K902E	-5.89	K902C	-5.13	K902C	-4.77	K902C	-6.52
	K902H	-5.55	K902S	-5.12	K902N	-4.90	K902N	-6.69
			K902N	-5.30	K902S	-4.77	K902S	-6.51
			K902E	-7.84	K902E	-6.56	K902E	-6.63
			K902D	-5.33	K902H	-6.19	K902H	-6.38
			K902H	-5.31				
**P929**	P929W	-7.70	P929W	+	P929W	-7.08	P929R	+
	P929M	-6.87	P929M	+	P929F	-6.94	P929N	-6.02
	P929R	-7.24	P929R	-6.09	P929M	-1.24	P929E	+
	P929N	-7.37	P929K	-3.93	P929R	-1.20		
	P929D	-7.26			P929C	-1.20		
					P929N	-1.20		
					P929E	-1.27		
**D945**	D945W	+	D945W	+	D945W	-1.88	D945W	-3.62
	D945R	-7.63	D945R	-7.00	D945R	-7.09	D945R	+
**S947**	S947W	-7.51	S947W	-6.98	S947W	-1.57	S947W	-7.67
	S947F	+	S947F	-6.99	S947F	-1.79	S947F	-7.62
	S947Y	-7.51	S947Y	-6.71	S947Y	-1.59	S947Y	-7.91
	S947L	+	S947M	+	S947I	-1.59	S947M	+
	S947I	+	S947R	-7.04	S947V	-1.59	S947R	+
	S947M	+	S947Q	+	S947M	-1.58	S947Q	+
	S947Q	+	S947N	+	S947R	-1.59	S947N	-5.24
	S947R	-7.50	S947K	-6.85	S947C	-1.57	S947K	-8.04
	S947N	+	S947E	+	S947Q	-1.60	S947E	+
	S947K	-7.49	S947D	+	S947N	-1.58	S947D	-5.21
	S947E	+	S947H	+	S947K	-1.58	S947H	-5.11
	S947D	+			S947E	-1.58		
	S947H	+			S947G	-1.57		
					S947D	-1.57		
					S947H	-1.59		

"+" denotes the lower affinity/positive value.

Table S3. Protein-ligand interaction energy as well as interacting residues (pi and H-bond) identified for all-natural variants obtained from the CDOCKER docking program in Discovery Studio Client v18.1.

Compound	Wild type	Mutated						
		Arg631		Tyr717	Asp868		Asp869	
		R631Q	R631P	Y717G	D868N	D868K	D869R	D869S
16A	-38.25	-37.43	-37.23	-37.93	-39.68	-35.34	-40.09	-39.79
	Tyr717, Arg631, Gly932, Cys931	Tyr717, Lys902, Cys931	Lys902	Arg631, Lys902	Lys626, Arg631, Lys710, Lys902	Lys626, Arg631, Lys710, Lys868, Lys902	Arg631, Tyr717, Arg869	Lys626, Arg631, Lys710, Lys902
4	-36.92	-37.28	-35.64	-41.97	-34.23	-37.01	-35.62	-37.93
	Tyr717, Arg631, Asp868	Lys902	Asp868	Arg631, Lys902	Arg631	Phe568, Arg631, Lys868, Lys902	Tyr717	Lys626, Arg631, Lys902
C_9i	-38.56	-37.50	-37.23	-40.86	-37.73	-38.98	-37.11	-42.09
	Tyr717, Arg631, Asp868, Gln833	Lys710, Tyr717, Cys931	Tyr717, Gln833, Asp868	Arg631, Lys710, Asp868, Cys931	Arg631, Arg865, Val867	Lys626, Arg631, Gly715, Lys868	Lys626, Arg631, Arg669, Val867	Arg631, Lys710, Tyr717, Lys902
C_9k	-39.96	-40.15	-40.85	-41.12	-38.78	-40.23	-41.51	-39.56
	Tyr717, Arg631, Asp868	Tyr717	Tyr717	Arg631, Lys902, Asp868, Gly717	Arg631,Gly715, Tyr717, Asn868	Arg631, Lys868	Phe568, Arg869, Cys931	Arg631

Table S4. Protein-ligand interaction energy as well as interacting residues (pi and H-bond) identified for the mutations of 16 residues that affected ligand affinity in MOE analysis, obtained from the CDOCKER docking program in Discovery Studio Client v18.1.

Comp.	Wild type	Mutated										
		K626			R631		K710		D712		A716	
		K626M	K626Q	K626R	R631E	R631S	K710E	K710Y	D712A	D712C	A716C	A716S
16A	-38.25	-35.35	-33.71	-33.11	-32.91	-34.76	-36.96	-38.44	-38.87	-38.32	-35.76	-34.90
	Tyr717, Arg631, Gly932, Cys931	Tyr717, Arg631	Arg631, Tyr717	Tyr717, Arg631	Lys626	Lys626, Lys902	Lys626	Phe568, Arg631, Tyr717	Arg631, Lys626	Tyr717, Arg631, Phe568	Arg631, Lys710, Lys902	Arg626, Gln833, Lys902
4	-36.92	-42.50	-42.32	-32.37	-33.21	-33.52	-35.28	-33.33	-35.42	-33.43	-35.54	-32.42
	Tyr717, Arg631, Asp868	Arg631, Lys902	Arg631, Gln833	Tyr717	Lys626	Lys626, Lys710, Asp868, Lys902	Arg631, Tyr717, Gln833	Lys626, Tyr717	Arg631, Phe568, Asp868	Lys626	Asp868	Arg631
C_9i	-38.56	-38.58	-39.14	-41.14	-39.11	-35.60	-37.88	-39.58	-38.46	-37.67	-39.52	-39.50
	Tyr717, Arg631, Asp868, Gln833	Tyr717, Arg631, Asp868	Arg631	Arg626, Arg631, Tyr717, Asp868	Arg669	Phe568, Lys626, Lys710, Lys902	Phe568, Arg631, Tyr717	Lys626, Arg631, Asp869	Lys710, Arg631	Arg631	Arg631, Asp868	Arg631, Asp868
C_9k	-39.96	-39.47	-38.37	-39.32	-39.22	-38.30	-41.03	-38.78	-38.75	-41.26	-39.45	-41.05
	Tyr717, Arg631, Asp868	Tyr717, Arg631, Asp868	Tyr717, Arg631, Asp869	Tyr717, Arg631	Lys626, Gly715, Asp868	Phe568, Lys626	Phe568, Arg631, Tyr717	Phe568, Lys626, Arg631	Tyr717, Arg631, Phe568	Tyr717, Arg631, Phe568	Arg631, Asp868	Arg631, Lys710, Lys902

	Y717			D718	Q833		T839	V867	D868		D869	
	Y717F	Y717H	Y717R	D718L	Q833D	Q833H	T839R	V867C	D868T	D868V	D869A	D869G
16A	-35.89	-42.27	-34.40	-37.26	-36.49	-38.90	-41.25	-41.67	-40.83	-38.92	-37.90	-38.74
	Lys626, Lys902	Lys626, Lys710, Lys902	Arg631, Arg717	Lys626, Lys902	Lys626, Arg631, Lys710, Lys902	Lys626, Arg631, Lys710, Lys902	Lys626, Arg631, Lys710, Lys902	Lys626, Arg631, Lys902	Lys626, Arg631	Lys626, Arg631	Lys626, Arg631, Lys902	Lys626, Arg631
4	-35.90	-36.19	-39.35	-34.78	-34.77	-36.06	-34.47	-35.06	-36.71	-36.74	-36.00	-35.37
	Lys626, Lys710, Lys902	Lys902, Cys931	Lys626, Lys710, Lys902	Lys902, Cys931	Lys710	Lys626, Arg631, Tyr717	Lys626, Arg631, Tyr717	Arg631, Tyr717, Gln833	Arg631	Lys626, Arg631	Tyr717, Gln833	Arg631, Gln833
C_9i	-35.10	-32.83	-45.53	-38.56	-39.02	-35.04	-37.45	-37.35	-39.89	-36.60	-37.74	-39.81
	Lys626	Arg631, His717	Arg631, Lys902	Phe568, Arg631, Lys710, Lys902	Lys626, Arg631, Tyr717, Asp833	Arg631, Tyr717	Phe568, Arg631, Tyr717, Gln833	Arg669, Cys842	Lys626, Arg631, Gly715, Tyr717, Asp869	Arg669	Phe568, Arg631, Tyr717, Asp868	Phe568, Arg631, Gly715, Tyr717
C_9k	-37.11	-36.05	-43.92	-38.66	-39.97	-37.23	-38.45	-36.91	-38.22	-41.13	-39.97	-44.63
	Phe717, Asp868, Lys902	Thr839	Arg631, Arg717, Gln833	Lys626, Tyr717	Arg631, Lys710, Asp869, Lys902	Lys710, Asp869, Lys902	Arg631, Tyr717	Lys626, Thr839, Asp868	Arg631, Gln833	Phe568, Arg631, Tyr717	Phe568, Lys626, Arg631, Asp712, Lys902	Phe568, Arg631, Tyr717, Asp868

	K902		P929		D945	S947
	K902H	K902R	P929F	P929W	D945R	S947K
16A	-35.40	-38.38	-39.58	-39.67	-40.62	-40.15
	Arg631	Phe568, Arg631, Tyr717	Phe568, Lys626, Tyr717, Gln833	Lys626, Arg631, Lys902	Arg631, Tyr717, Lys902	Lys626, Arg631, Lys710, Lys902
4	-37.91	-35.19	-34.88	-35.13	-34.41	-33.23
	Lys626, Asp868	Tyr717, Asp868, Lys902	Phe568, Lys626, Lys902	Arg631, Trp929	Lys710, Lys902, Arg945	Lys626, Arg631
C_9i	-37.29	-37.65	-35.83	-37.97	-36.91	-35.75
	Arg631	Arg631, Lys710, Arg902	Phe568, Arg631, Tyr717	Phe568, Arg631, Lys902, Cys931	Arg631, Lys710, Asp869	Arg631, Tyr717
C_9k	-42.54	-41.13	-38.96	-37.85	-38.69	-37.53
	Phe568, Arg631, Tyr717	Phe568, Arg631, Tyr717	Lys626	Thr567, Phe568, Lys626, Arg631	Tyr717, Asp869, Lys902, Arg945	Arg631, Gly715, Tyr717

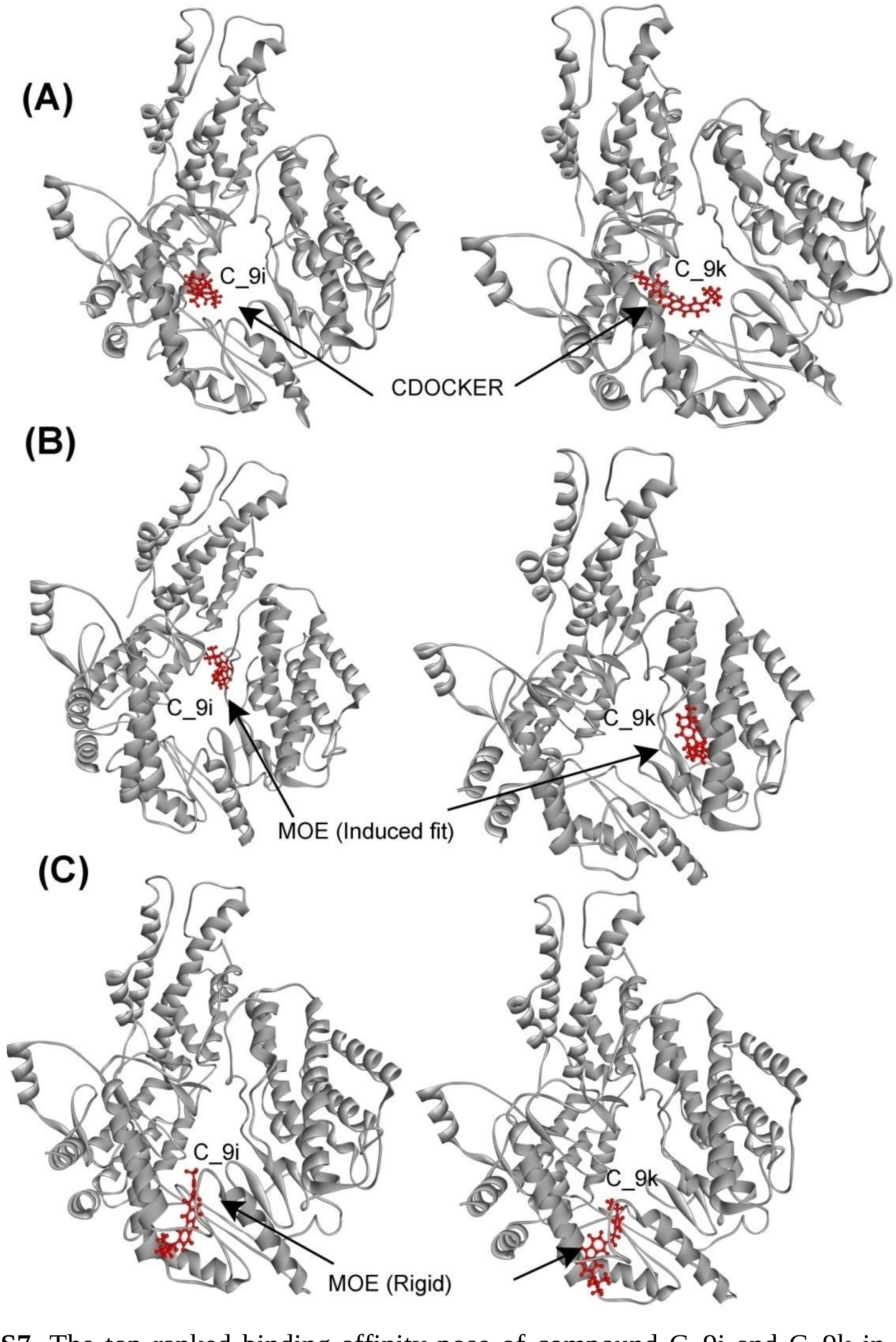

Figure S7. The top ranked binding affinity pose of compound C_9i and C_9k in different docking programs, CDOCKER, MOE rigid and induced fit docking. Each of the binding mode obtained from different docking programs was studied in complex with WT telomerase by MD simulation and for mutant models, the best docked conformation obtained from CDOCKER was taken as the starting structure for the MD run.

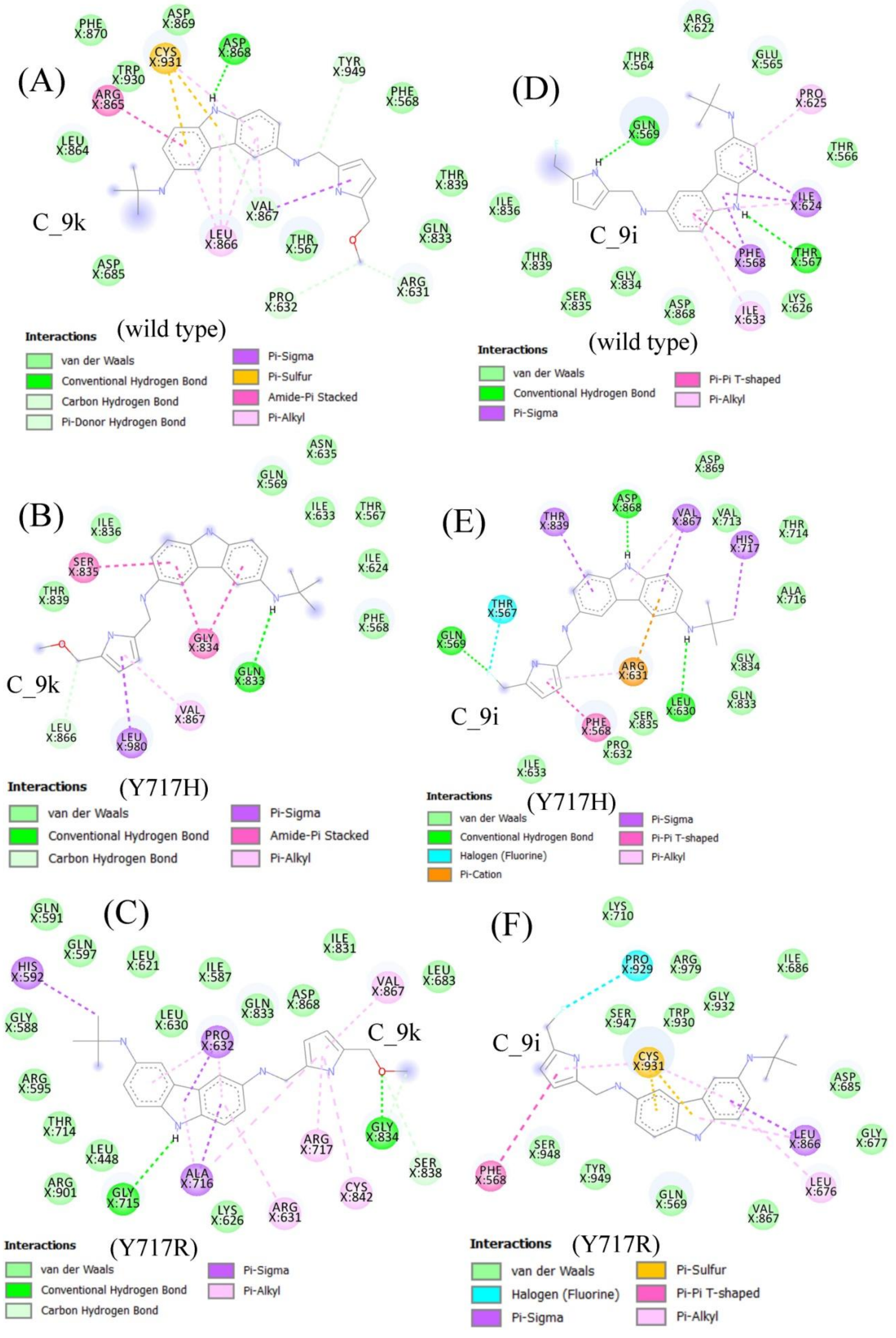

Figure S8. The 2D interaction diagrams showing the interaction pattern of the ligands C_9i and C_9k in all simulated models.

Table S5. The list of residues forming hydrogen bonds (H-bond) with an occupancy (%) describing protein-ligand interactions in simulated systems (CDOCKER binding mode).

**C_9i**		**C_9k**		**C_9i**		**C_9k**	
**Wild type**				**Y717H**			
**Residue**	**%**	**Residue**	**%**	**Residue**	**%**	**Residue**	**%**
Thr567	14.37	Asp868	15.47	Asp868	16.07	Gln569	6.49
Glu565	13.35	Cys931	11.88	Thr567	5.49	Thr839	4.20
Asp868	8.88	Asp869	3.19	Gln833	4.89	Gln833	2.79
Gln569	2.70	Asp685	2.99	Pro632	2.89	Asp637	2.50
Gly834	1.90			Gly834	2.10	Leu866	1.60
Pro625	1.50			**Y717R**			
Thr564	1.20			Asp945	12.87	Gly834	23.95
				Asp868	11.88	Gln833	11.38
				Cys931	8.48	Asp718	6.19
				Ser947	5.89	Asp868	5.79
				Asp869	4.19	Thr839	1.20
				Gln569	3.00		

Table S6. The list of residues forming hydrogen bonds (H-bond) with an occupancy (%) describing protein-ligand interactions in MD simulated systems with binding mode from MOE as starting points; induced fit and rigid docking, respectively.

**C_9i**		**C_9k**		**C_9i**		**C_9k**	
**Induced fit**				**Rigid**			
**Residue**	**%**	**Residue**	**%**	**Residue**	**%**	**Residue**	**%**
Lys570	31.44	Gln1008	12.48	Leu675	48.90	Arg865	20.16
Ser957	30.54	Leu980	3.79	Arg858	10.88	Arg865	15.87
Val977	10.98	His983	2.69	Ile857	9.88	Asn851	5.79
Gln569	8.58	Ser1045	1.40	Leu676	4.49	Gly674	1.20
Leu978	3.49	Gln1008	1.00	Leu863	2.40	Asp848	1.10
Leu958	2.99			Glu850	2.20		
Gln569	2.89			Gly677	1.50		
Tyr946	2.10			Arg859	1.00		
Ser947	1.40						

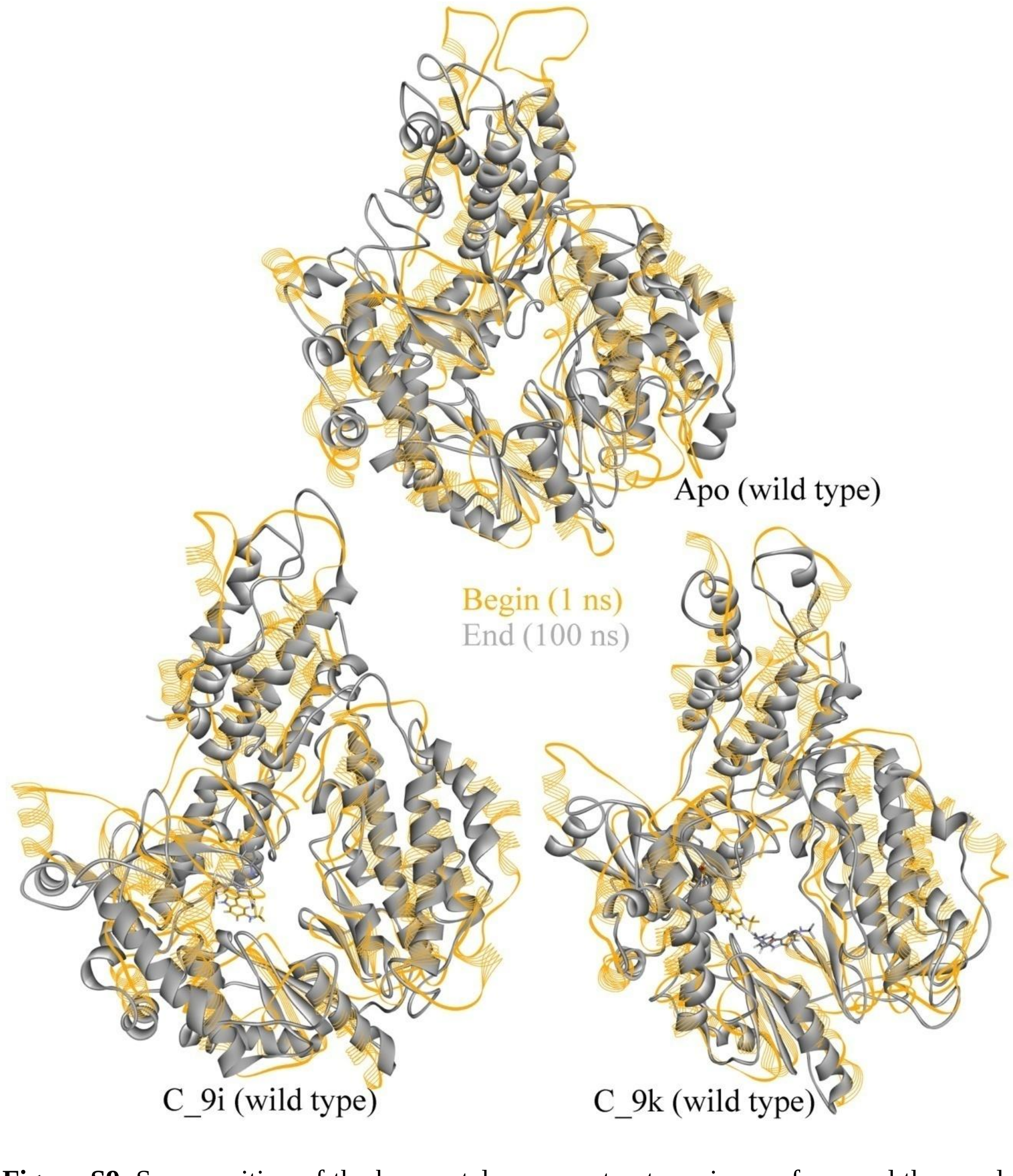

Figure S9. Superposition of the human telomerase structures in apo-form and the overlay of the binding mode of the inhibitors C_9i and C_9k to wild-type human telomerase model, obtained at the beginning and end of MD simulations.

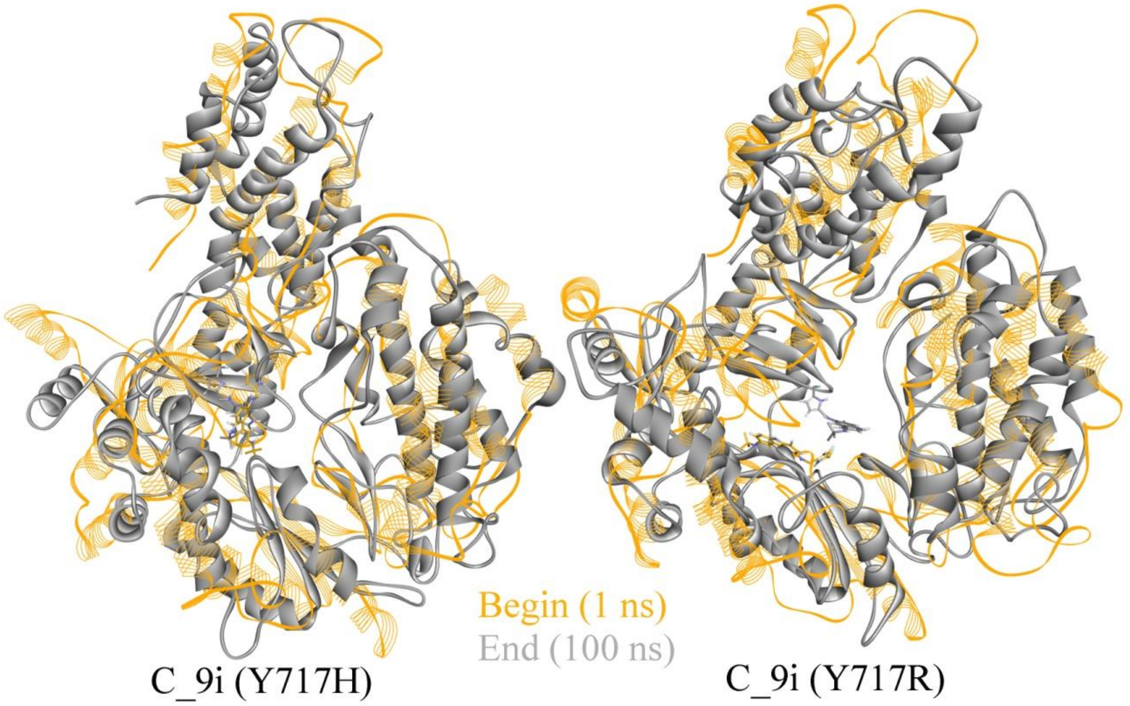

Figure S10. Overlay of the binding mode of inhibitors C_9i to the mutated human telomerase models (Y717H and Y717R) that were obtained at the beginning and end of the MD simulations.

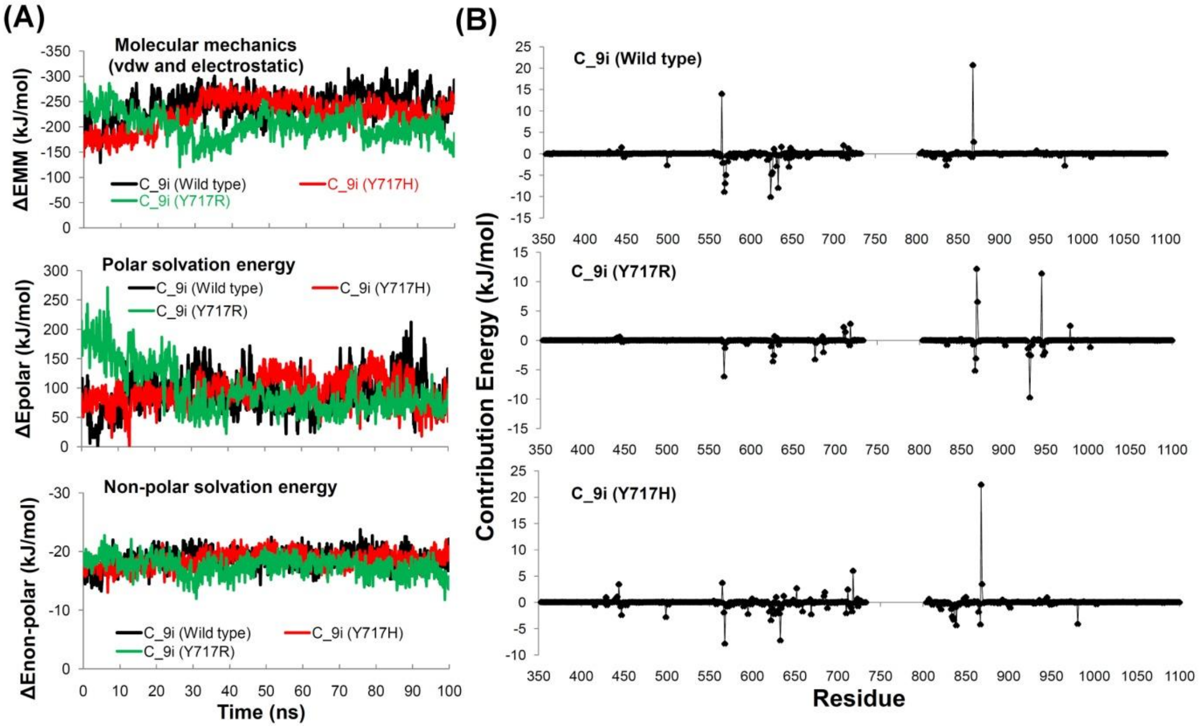

Figure S11. (A) Binding energy components calculated by MM-PBSA method for compound C_9i with telomerase (from MD of CDOCKER predicted starting binding mode).
(B) The contribution of each residue to the total MM-PBSA binding energy.

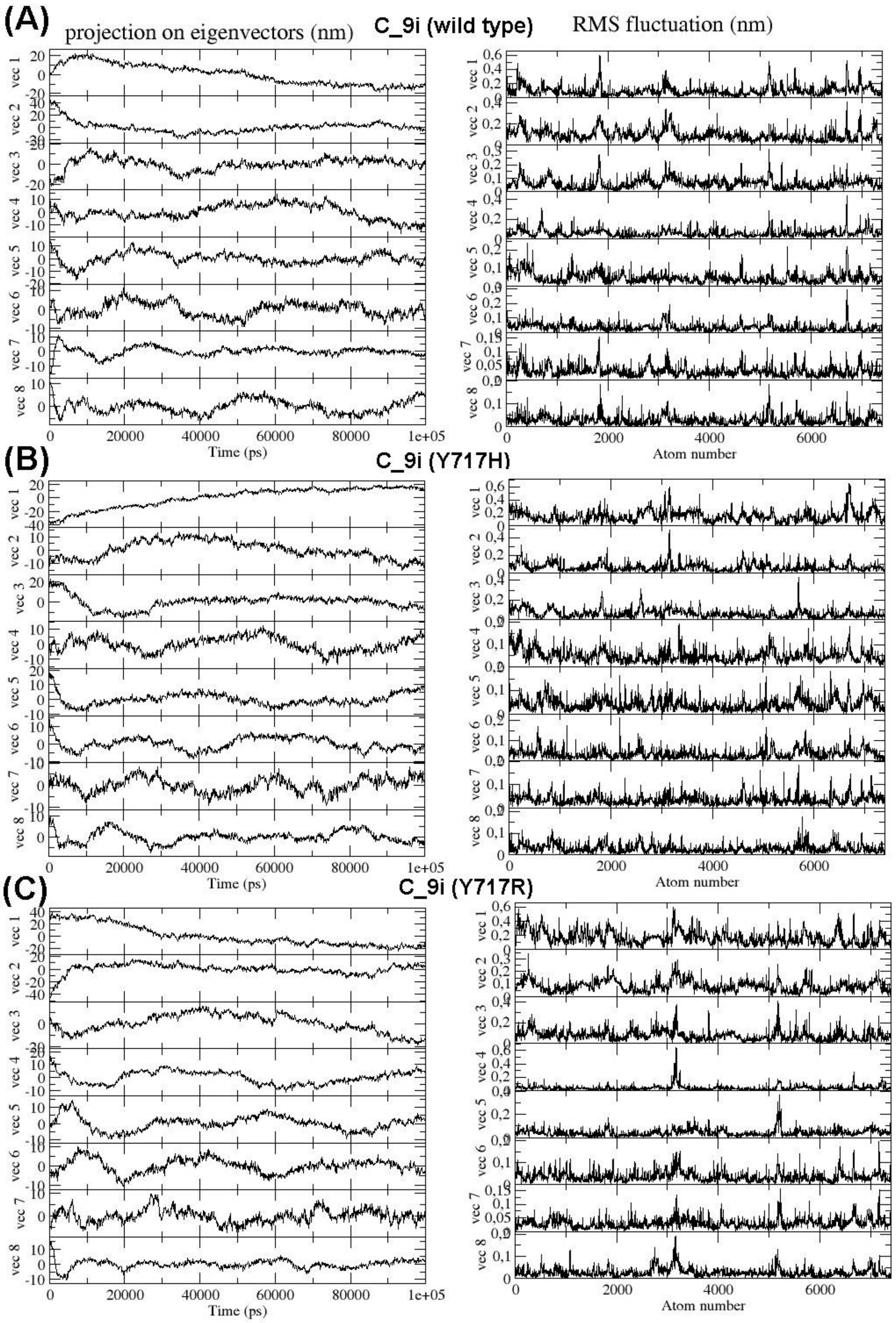

Figure S12. MD trajectory projection along all eigenvectors and the RMSF calculated along selected eigenvectors.

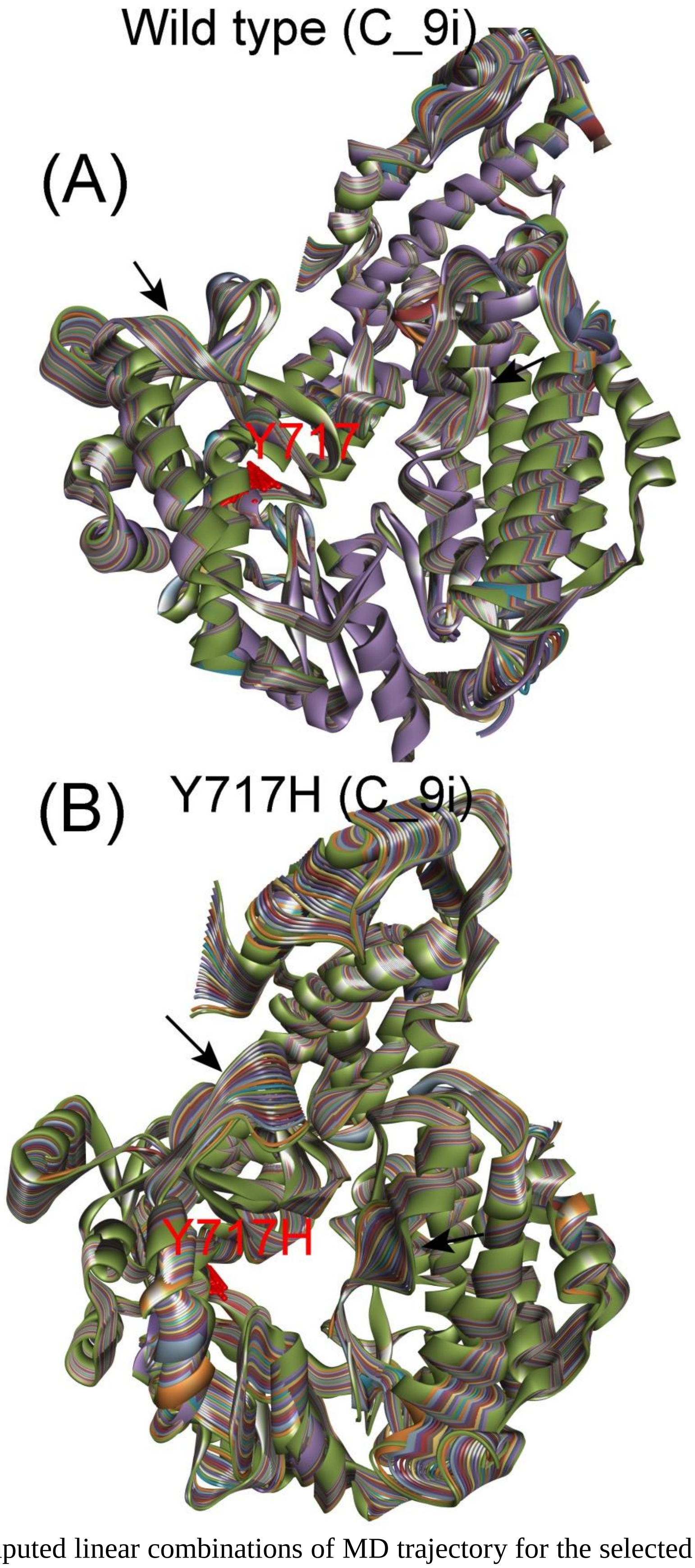

Figure S13. Computed linear combinations of MD trajectory for the selected eigenvectors_1, for human telomerase with ligand C_9i (wild type and mutation Y717H).
